# Assessing the utility of a sliding-windows deep neural network approach for risk prediction of trauma patients

**DOI:** 10.1038/s41598-023-32453-3

**Published:** 2023-03-30

**Authors:** Alexander Bonde, Mikkel Bonde, Anders Troelsen, Martin Sillesen

**Affiliations:** 1 Department of Organ Surgery and Transplantation, Copenhagen University Hospital, Rigshospitalet, Copenhagen, Denmark; 2 Center for Surgical Translational and Artificial Intelligence Research (CSTAR), Copenhagen University Hospital, Rigshospitalet, Copenhagen, Denmark; 3 Department of Orthopedics, Copenhagen University Hospital, Hvidovre, Denmark; 4 Institute of Clinical Medicine, University of Copenhagen, Copenhagen, Denmark

**Keywords:** Trauma, Risk factors

## Abstract

The risks of post trauma complications are regulated by the injury, comorbidities, and the clinical trajectories, yet prediction models are often limited to single time-point data. We hypothesize that deep learning prediction models can be used for risk prediction using additive data after trauma using a sliding windows approach. Using the American College of Surgeons Trauma Quality Improvement Program (ACS TQIP) database, we developed three deep neural network models, for sliding-windows risk prediction. Output variables included early- and late mortality and any of 17 complications. As patients moved through the treatment trajectories, performance metrics increased. Models predicted early- and late mortality with ROC AUCs ranging from 0.980 to 0.994 and 0.910 to 0.972, respectively. For the remaining 17 complications, the mean performance ranged from 0.829 to 0.912. In summary, the deep neural networks achieved excellent performance in the sliding windows risk stratification of trauma patients.

## Introduction

Traumatic injury remains the leading cause of morbidity and mortality among individuals under the age of 45 years^1^. In the US alone, trauma deaths have increased by 22.8%, from 2000 to 2010, with the largest increase seen in patients between 50 and 70 years of age^2^.

Although advances in trauma care are reflected in increased survival following major injury^3^, trauma is still associated with a significant risk of morbidity. Reports have indicated that up to 25% of patients admitted to an Intensive Care Unit (ICU) following trauma experience a post-trauma complication (PTC, e.g. infectious, organ failure or thrombotic events)^4^.

Care providers still rely on scoring systems like the Injury Severity Score (ISS), standard operating procedures (SOPs) as well as clinical gestalt^5^. The scoring systems often map linear associations of simplified data, and like SOPs, they fail to capture the dynamic changes of the clinical setting over time^6^. While researchers have investigated numerous technical approaches to risk prediction following both trauma and surgery^6–9^, the approaches often use single timepoint predictions, which creates a setting where the model precision will become increasingly unstable as the clinical treatment trajectories progresses.

Trauma patients generate a plethora of biomedical data as they proceed through clinical trajectories from the point of injury. The ideal predictive model in trauma care would thus not only use data obtainable at a single timepoint, but rather be adaptable to the influx of biomedical data as it is generated. Such incremental data is, however, often too complex for human-level aggregated decision making.

Recent developments in the field of machine learning, specifically within deep learning, have shown promise in the task of risk stratification through analyses of complex patient data flows^10–12^. Whether these networks could be of value in the risk stratification of trauma patients as they advance through the clinical treatment trajectories, is currently unknown and constitutes the focus of this study. We hypothesized that deep neural networks could provide accurate risk stratification of the trauma patients in terms of PTCs at distinct time points.

## Methods

### Data source

We used data from the American College of Surgeons Trauma Quality Improvement Program (ACS TQIP) 2017 data set, including 997,970 trauma cases. Patients with a missing hospital length of stay (n = 18,962) were excluded.

**Figure 1:**
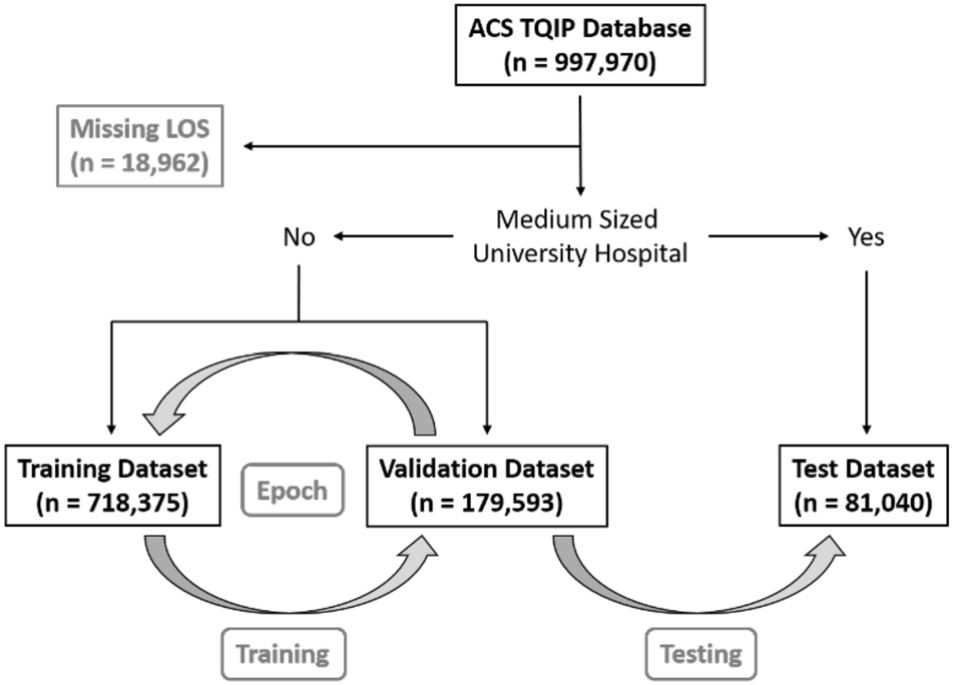
A graphical overview of patient selection and group allocation. This study is based on the ACS TQIP database from 2017 which included a total of 997,970 trauma cases. Only patients with a missing hospital length of stay were excluded (LOS, n = 18,962). Patients that were treated at medium-sized (bed size = 201–400) university hospitals were reserved for final performance evaluation as the test dataset (n = 81,040). The remaining 897,968 cases were randomly split into a training dataset (n = 718,375) and a validation dataset (n = 179,593). The grey arrows indicated the training- and validation loop, followed by final model testing. One “epoch” has passed, when a model has seen been trained on all datapoints in the training dataset once. After each epoch, the performance of the newly trained model is calculated on the validation dataset. We trained all our models for a total of five epochs, before calculating the final performance on the test dataset.

To test the portability of the models, we created a test set with institutions that were external to the in-sample (train + validation) data. This test set consisted of medium-sized (bed size = 201–400) university hospitals (n = 81,040). The remaining dataset (n = 897,968) was randomly split into a training dataset (n = 718,375) and a validation dataset (n = 179,593). A graphical overview of patient selection and group allocation is presented in Fig. 1. The validation set was used to validate performance on in-sample data, while the test set was used to test performance on out-of-sample data. We used the standard fast.ai hyperparameters for dropout, embeddings, weight decay, and number of epochs, as proposed by the former President and Chief Scientist of Kaggle and co-founder of fast.ai, Jeremy Howard^13^.

Data were handled in line with the TQIP data-user agreement and access was granted by the ACS TQIP. Requirements for local IRB approval for handling the de-identified TQIP dataset was waived according to national law in Denmark. As permission for this type of study is governed by national law and thus regulated above the level of local ethics committees, the involvement of ethics committees is not required for neither reviewing nor waiving permission for such studies locally. All methods were performed in accordance with relevant guidelines and regulations.

### Input variables

**Figure 2:**
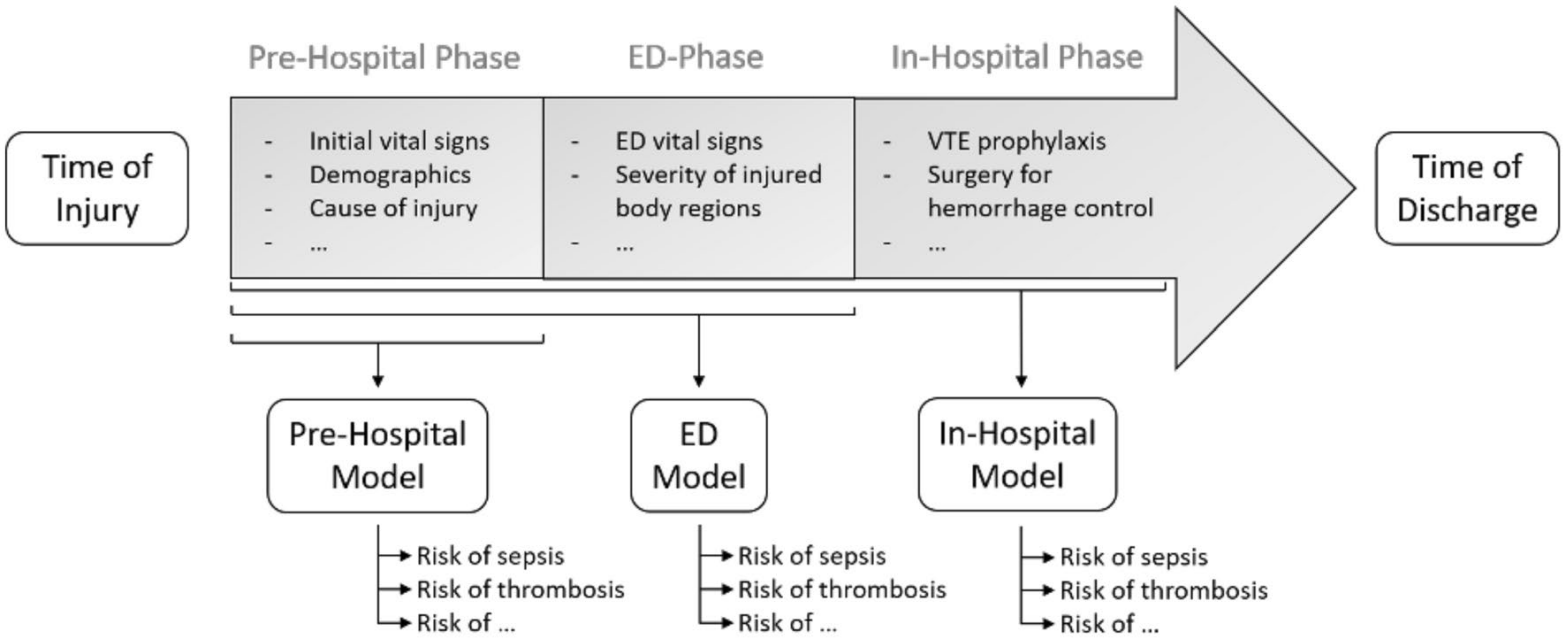
A graphical overview of a typical trauma patient treatment trajectory. An overview of a typical treatment trajectory for trauma patients. The timeline is divided into a time of injury, a pre-hospital phase, an emergency department (ED) phase, an in-hospital phase, and a time of discharge. As evident, an increasing amount of biomedical data becomes available, as patients pass through the trajectory. The Pre-Hospital model is trained on variables like to be available in the pre-hospital phase, such as initial vital signs at the scene of injury, demographics, and cause of injury. The ED Model includes additional input variables likely to be available early in the ED-phase, such as vital signs in the ED and the severity of each injured body region. The In-Hospital Model includes additional input variables likely to be available within the first day of hospitalization, such as the type of  Venous Thromboembolism (VTE) prophylaxis and the type of surgery for hemorrhage control*.*

We created three models: one for the pre-hospital setting (Pre-Hospital Model), one for the emergency department (ED Model) and one for the second day of hospitalization (In-Hospital Model). A graphical overview of the three models is presented in Fig. 2.

Input variables for all three models, are presented in the Online Supplementary Material (Tables 1A–1C).

The Pre-Hospital Model was trained on variables likely to be available in the pre-hospital setting, while the ED Model included additional input variables collected within the first hours after arrival in the ED. The severity of each injured body region was derived from the first- and last numbers of the AIS code).

For the In-Hospital Model, we included additional variables that were recorded within the first day of hospitalization.

Data are presented as medians [interquartile range] or percentages where appropriate.

### Preprocessing of input variables

We one-hot encoded the severity of each injured region and used entity embeddings to create vectors for all categorical variables. The concept of entity embeddings maps the categorical variables vectors in an n-dimensional space, where variables with shared features are mapped closer together than unrelated variables, thus enabling the model to learn similarities between these variables. As an example of this, falls as the primary cause of injury would be mapped closer in the embedding matrices as opposed to codes indicating a motor vehicle accident. The embedding layers were created by randomly initializing a vector to each of the categorical values. The length of each vector was based on the following formula: min(600, round(1.6 * n_cat ** 0.56)), as implemented in the fast.ai library. In this formula, n_cat represents the cardinality of the category. Cardinalities ranged from two for “fatal upper extremity injury” (no patients had this AIS code, and categorical values only included 0 and missing) to 1995 for the “primary external cause of injury”. This resulted in vector lengths between 2 and 113. Vectors for each categorical variable were stacked into embedding matrices, where the number of rows was equal to the cardinality. This resulted in embedding matrices with parameters ranging from 4 (cardinality of 2 × vector length of 2) to 225,435 (cardinality of 1995 × vector length 113). Parameters in these matrices were trainable and were updated along with the linear layers.

Missing values were treated differently for categorical and continuous data. For categorical data, missing values were kept as distinct categories within each variable. For continuous data, the missing values were replaced with the median of the group, with a new binary column indicating whether a variable was missing or not. Then, continuous variables were normalized by subtraction of the mean and division by the standard deviation.

### Output variables

Output variables for all three models, along with summary statistics for the training-, validation-, and test datasets, are presented in the Online Supplementary Material (Table 1D). Mortality was split into early- and late mortality. Early mortality was defined as either an ED discharge disposition of “deceased/expired” with a length of stay (ED or total) of one day, or a hospital discharge disposition of “deceased/expired” with a total hospital length of stay of one day. As models would never be used on dead patients, we excluded patients with early mortality from the In-Hospital Model test dataset. Late mortality was defined in the same way, but with a length of stay of more than one day.

All three models were trained to predict late trauma deaths, whereas only the Pre-Hospital and the ED Models were trained to predict early trauma death. For the In-Hospital Model, early mortality was changed from an output- to an input variable.

### Neural network architecture

**Figure 3:**
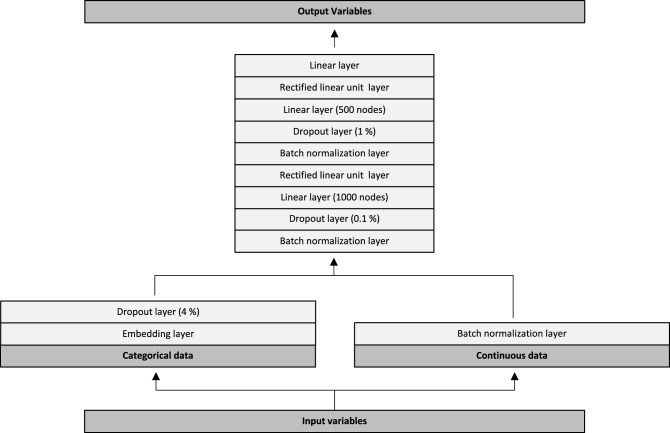
Neural network architecture. Input variables were split into categorical- and continuous data. Categorical data were passed through trainable embedding matrices followed by a 4% dropout layer. Continuous variables were passed directly through a batch normalization layer. Subsequently, values from the categorical and continuous trajectories were combined into another batch normalization layer. This was followed by a dropout of 0.1%, a linear layer with 1000 nodes and a rectified linear unit (ReLU) layer. Data were then passed through a batch normalization layer, a dropout of 1%, a linear layer with 500 nodes and another ReLU layer. Lastly, the data was passed through a linear layer with 19 outputs, one for each of the output variables (18 outputs for the In-Hospital Model).

The neural network architecture is graphically depicted in Fig. 3. Input variables were initially split into categorical- and continuous data. Categorical data were passed through trainable embedding matrices followed by a 4% dropout layer. Continuous variables were passed directly through a batch normalization layer. Subsequently, values from the categorical and continuous trajectories were combined into another batch normalization layer. This was followed by a dropout of 0.1%, a linear layer with 1000 nodes and a rectified linear unit (ReLU) layer. Data were then passed through a batch normalization layer, a dropout of 1%, a linear layer with 500 nodes and another ReLU layer. Lastly, the data was passed through a linear layer with 19 outputs, one for each of the output variables (18 outputs for the In-Hospital Model). We used the Adam optimizer combined with a flattened binary cross entropy loss function. We trained all models for five epochs, with a batch size of 1024, a weight decay of 0.2 and a learning rate of 3e−3. We evaluated model performance for each of the 19 output variables on both the validation- and the test dataset. Performance metrics included the area under the receiver operating characteristic curve (ROC AUC), with 95% confidence intervals, as well as the Brier score. Confidence intervals were calculated using the formula: AUC ± standard error × 1.96. The standard error of the AUC was calculated as described by Hanley and McNeil^14^.

The only differences in architecture between the three models, were the number of embedding matrices-, input variables and output variables. The models were developed with 986,969, 1,388,133 and 1,407,857 trainable parameters and with 40, 143 and 147 embedding layers, respectively. The remaining parts of the neural networks were similar across all three models.

### SHAP feature importance

We employed a game theoretical approach (Shapley Additive Explanations, SHAP)^15^ to gain insight into the relative importance of each input feature. The average of the marginal contributions across all permutations (Shapley Values) was calculated for 10,000 randomly selected patients from the test dataset. These values were used to rank order the top 20 most important data points for final risk prediction (Shapley Summary Plots)^16^ using SHAP v0.35.0^15^.

### Random forests

For model comparison, we created 56 random forests, one for each of the output variables, with each of the three input options (Pre-Hospital, ED, and In-Hospital). Variables did not include entity embeddings, as this is a specific feature of neural networks. The remaining preprocessing steps were the same as previously described.

Each forest included 100 trees, each trained on a sample of 200,000 rows, with 50% of all columns sampled at each split point. The depth of each tree was limited with a requirement of at least five samples per node. For each forest, we calculated feature importance to rank data points relevant for each of the output variables. Feature importance was based on Scikit-learn’s decrease of mean impurity.

### Implementation

The neural networks were implemented using Python v. 3.8.3, PyTorch v. 1.6.0^17^ and Fastai v. 2.0.11^18^. We calculated the Receiver Operator Characteristics Area Under the Curve (ROC AUCs) using the Scikit-learn v. 0.23.1^19^. We employed a game theoretical approach (Shapley Additive Explanations), to estimate the significance of each input feature towards final risk prediction^16^, using SHAP v 0.35.0^15^. Random forests with associated feature importance plots were implemented using Scikit-learn v. 0.23.1^19^.

## Results

In the overall dataset, 60% were male, the median age was 49 [26–69] and 9.9% were pediatric cases (age under 16 years). The median ISS was 8 [4–10]. A total of 12,651 patients suffered early deaths (within one day of injury, 1.3%) and 19,574 late deaths (2.0%). For the PTCs included in our prediction models, we found an overall morbidity rate of 3.3%. The median length of stay was 3 days [2–6]. Detailed characteristics of the study populations in each of the training, the validation, and the test datasets are presented in the Online Supplementary Material Tables 1A, 1B and 1C.

### Performance

**Figure 4:**
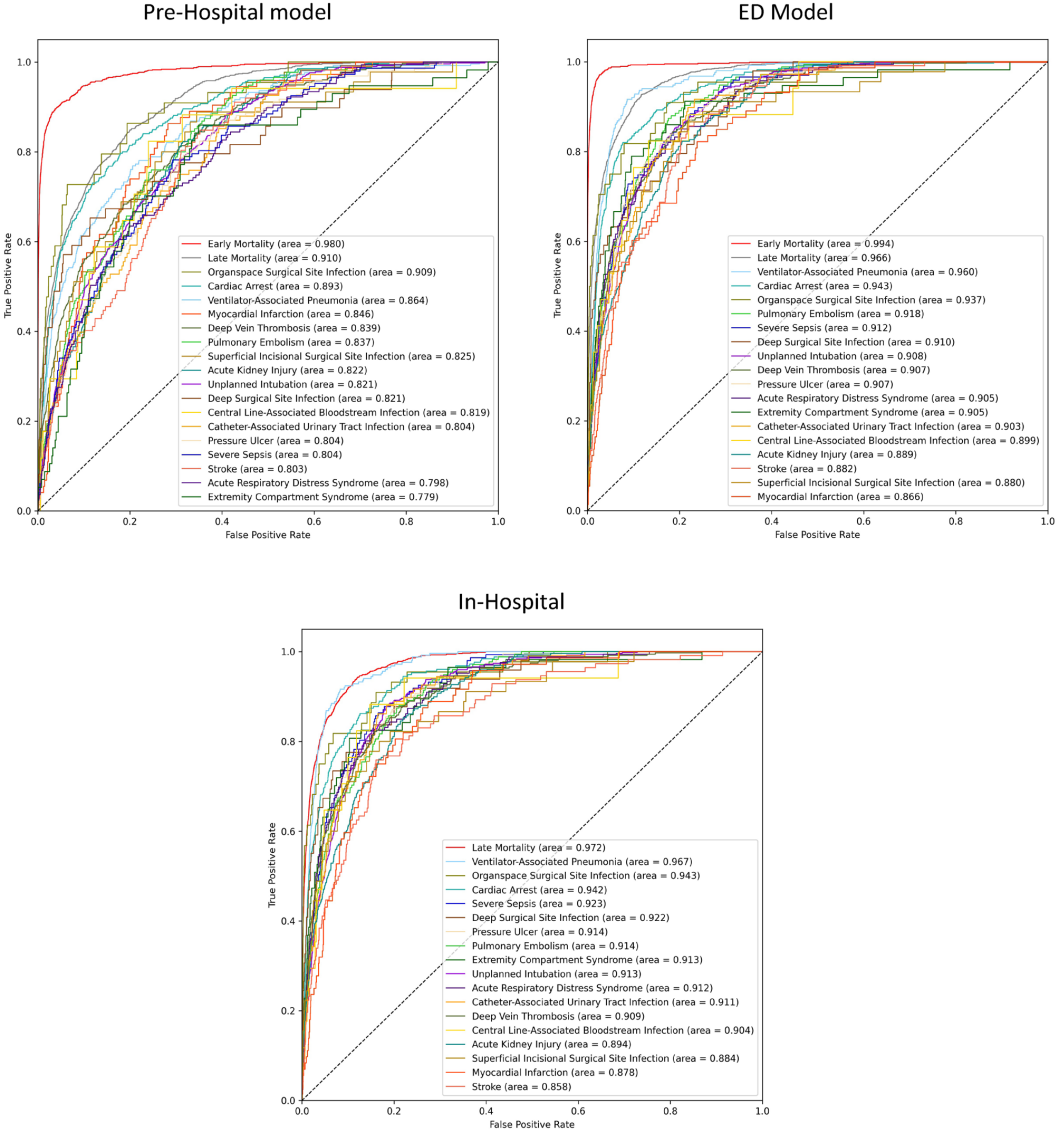
Receiver operating characteristics for each of the three models. The areas under the curves (area) are ordered after descending scores. Early Mortality = Mortality within the first day of hospitalization, Late Mortality = Mortality after the first day of hospitalization.

Performance metrics for each of the three models, both on the validation- and the test datasets, are presented as ROC AUC scores with 95% confidence intervals in the Supplementary Table 2, and as a Brier score in the Online Supplementary Table 3. ROC curves for each of the three models, are presented in Fig. 4. As the patients moved through the treatment trajectories, with incremental data becoming available, we saw a corresponding increase in model performance. In the following section, ROC AUC scores on the test dataset are summarized.

The Pre-Hospital Model predicted early death with a test ROC AUC of 0.980 and late death with a score of 0.910. These numbers increased for the ED Model, which achieved near-perfect results, with scores of 0.994 and 0.966, respectively. The In-Hospital model exhibited the best predictive ability for late mortality, with a ROC AUC of 0.972.

For the remaining PTCs, the Pre-Hospital Model achieved an average test ROC AUC of 0.829, ranging from 0.779 for extremity compartment syndrome to 0.909 for organ space surgical site infection. For the ED Model, the average score increased to 0.908, ranging from 0.866 for myocardial infarction to 0.960 for ventilator-associated pneumonia. Overall, we also noted an increase between the ED Model and the In-Hospital Model, where the average score was 0.912, ranging from 0.858 for stroke to 0.967 for ventilator-associated pneumonia.

### SHAP feature importance

**Figure 5:**
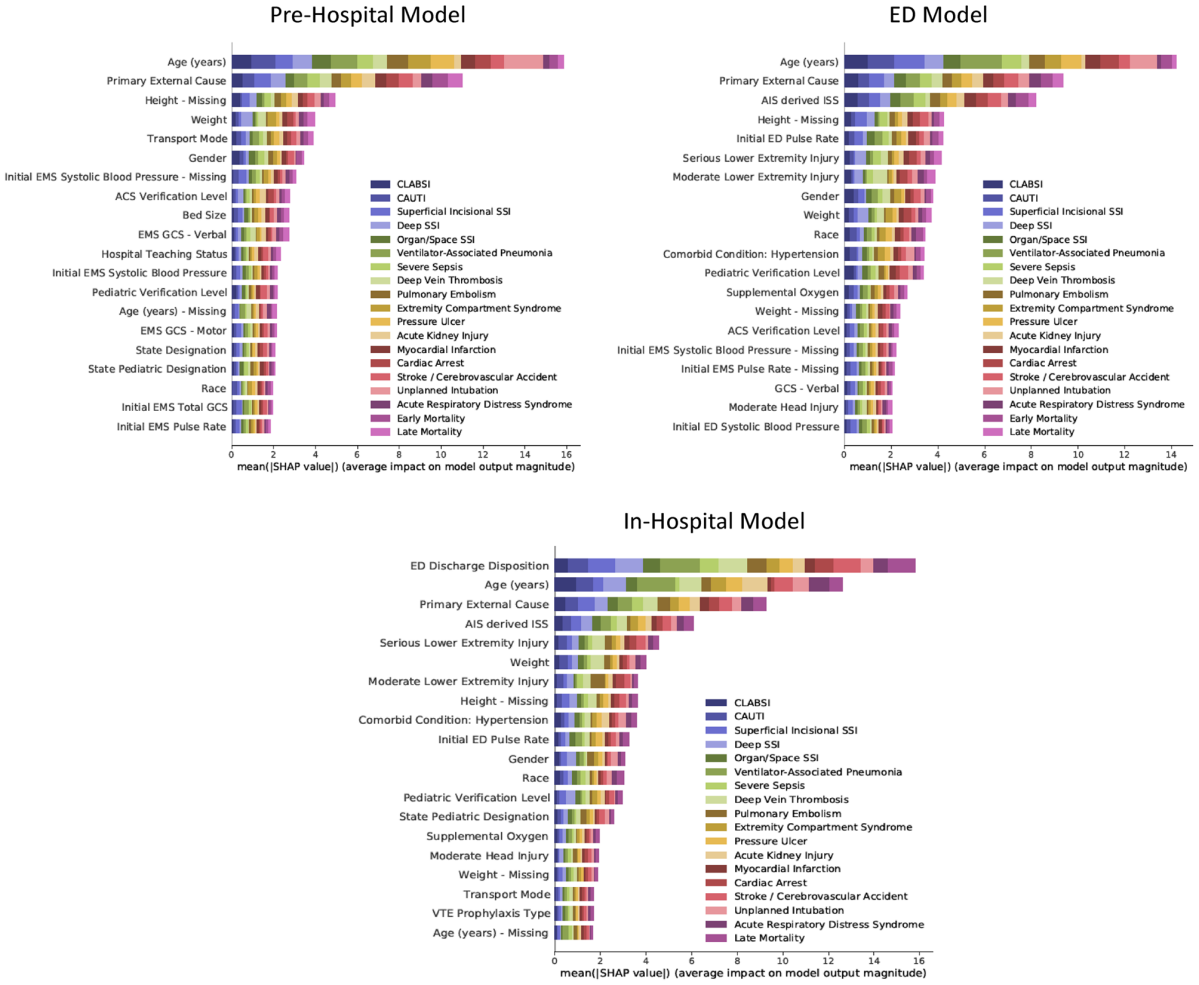
The permutation importance for the top 20 most important input features for each of the three models. The values on the x-axis indicate the average impact on model output magnitude based on Shapley Additive Explanations (SHAP). The features on the y-axis are arranged so that the input features with the largest average impact on model output magnitude are placed in the top. The horizontal bars are color-coded to match the output variables. The larger the colored area the more impact the model has on the corresponding. *CLABSI* Central line-associated bloodstream infection, *CAUTI* Catheter-associated urinary tract infection, *SSI* Surgical site infection, *Early Mortality* Mortality within the first day of hospitalization, *Late Mortality* Mortality after the first day of hospitalization, *EMS* Emergency Medical Services, *ACS* American College of Surgeons, *GCS* Glasgow Coma Scale, *AIS* Abbreviated Injury Scale, *ISS* Injury Severity Score, *ED* Emergency Department, *VTE* Venous thromboembolism, *na* Missing.

SHAP summary plots are presented in Fig. 5. The color codes of the plots illustrate the relative importance of an input feature towards the prediction of each PTC.

For the Pre-Hospital Model, the most important input feature was the age of the patient, followed by the primary external cause of injury. When looking at individual output variables, age appeared disproportionally important for the prediction of unplanned intubation and less important for the prediction of acute kidney injury. Although the primary external cause of injury was less important than age overall, it was more important when predicting certain output variables such as acute respiratory distress syndrome, early mortality, and late mortality. Interestingly, some missing variables ranked among the 20 most important input features, indicating that the data were not missing at random.

For the ED model, the most important additional features were the injury severity score and the first measured pulse rate in the emergency department. Hypertension appeared to be the most important comorbidity.

For the In-Hospital Model, the emergency department discharge disposition became the most important input feature. When compared to the age of the patient, this input feature was especially important for the prediction surgical site infections. Age was still more important when predicting selected outputs such as myocardial infarction, cardiac arrest, and stroke.

### Random forests algorithms—performance

Performance metrics for the random forest algorithms are presented as ROC AUC scores with 95% confidence intervals in the Online Supplementary Material Table 4, and as a Brier score in the Online Supplementary Material Table 5. When compared to neural networks, random forests performed inferiorly on all output variables. The best results were seen on mortality, where the models achieved ROC AUCs between 0.867 and 0.992. For the remaining outputs, the mean ROC AUC on the test dataset was 0.698 for the Pre-Hospital Model, 0.838 for the ED Model, and 0.851 for the In-Hospital model.

### Random forests algorithms—feature importance

Feature importance for each of the 56 random forest models are presented in the Online Supplementary Material Fig. 1A–C.

When predicting early mortality with the Pre-Hospital Model, prehospital cardiac arrest was the most important input feature. Missing weight and height ranked as the 4th and 8th most important input features.

For the ED Model, the initial blood pressure and pulse rate in the ED ranked highly for predicting early mortality. Critical head injury ranked as the most important input feature for late mortality. When compared to the neural networks, the primary external cause of injury had a lower relative importance for almost all outputs.

For the In-Hospital Model, the extra input features only ranked among the 10 most important inputs for two outputs (cardiac arrest and late mortality).

## Discussion

In this study, deep neural networks were trained on structured electronic health care data for accurate risk stratification of trauma patients, as they advance through clinical treatment trajectories. We demonstrate that additive data increase model performance as assessed through a sliding-windows approach. When predicting the risk of PTCs at distinct time points, our models achieved test ROC AUC scores between 0.779 and 0.994. As the test dataset consisted of institutions that were external to the in-sample data (train + validation), the results reflect performance that could be expected from institutions that the models have never seen before. After training, validating, and testing the networks, models proved valuable for deciphering and ranking data points relevant for clinical outcomes.

The Pre-Hospital Model included input variables that were likely to be available in the prehospital setting. The only injury-related inputs were the primary causes of injury, and the criteria for transport to a trauma center (penetrating injury, pelvic fracture etc.). Interestingly, this model still performed well, indicating that deep learning could be of value in the acute setting. However, performance increased significantly with the additional input variables for the ED Model. Especially the addition of detailed injury characteristics, derived from the abbreviated injury scale, appeared to increase performance. If this input variable was excluded from the ED model, the average ROC AUC dropped from 0.908 to 0.886.

Other studies have reported cross-validation prediction metrics with ROC AUCs between 0.82 and 0.97 for death, 0.82–0.86 for venous thromboembolic events, and 0.71–0.83 for acute respiratory distress syndrome^20^, while another study on the TQIP dataset reported mortality ROC AUC prediction of 0.941, VTE predictions ROC AUC of 0.734 and acute respiratory distress syndrome ROC AUC of 0.786^8^.

Other machine learning projects have found mixed results for the risk stratification of trauma patients. These include studies on severe sepsis and septic shock (random forest classifier, ROC AUC = 0.88)^20^, pulmonary emboli (random forest classifier, ROC AUC = 0.71)^21^, acute respiratory distress syndrome (L2-logistic regression, ROC AUC = 0.81)^20^ and acute kidney injury (random forest classifier, ROC AUC = 0.84)^20^. Machine learning based on additive data for sliding windows risk prediction has previously been assessed for the prediction of mortality after traumatic brain injury^22^. In this study, the authors also found an increase in model performance from 0.67 on day 1 to 0.81 on day 5.

Although a direct comparison of performance remains challenging, as methods and data varied between studies, the results collectively indicate that neural networks appear to have a performance advantage over both previously published machine learning- as well as legacy approaches. While it remains a challenge to decipher the reason why this approach might be superior to other methods, the results mirror recent findings from surgical patients, where a deep learning approach was also superior to random forests^12^.

While the models presented here are not dynamic in the sense that they are able to ingest available time point, the results Our results indicate that future risk prediction models in both trauma and other clinical settings could benefit from using a dynamic approach, where predictions are based on available data at the time, rather than on a static input dataset.

While the fact that ROC–AUCs in this study increased as more data became available supports this notion, it is obviously also important to underline that simple clinical gestalt would also exhibit increasing predictive precision as more clinical data becomes available, as is exemplified by the ability to predict adverse outcomes in a patient deteriorating after trauma resuscitation. While the true measure of the model’s usability should be benchmarked against seasoned clinical judgement, the prediction of distinct PTCs such as VTE or infection that can be used for a precision medicine approach remains challenging for even veteran clinicians. In line with this, it is important to underline that predictions made by any modelling approach should not only be externally validated, but also have direct clinical implication. As such, while predicting mortality risk may not alter the treatment course in a young patient, where maximal treatment efforts are instigated, predicting amendable events such as VTE may be used to alter the treatment course.

While analyzing the full extent of complex clinical data by neural networks as this becomes available may be superior to previously published approaches, the complexity of the input data necessitates direct implementation into Electronic Health Records (EHR) for automated data extraction, an approach that has previously been demonstrated to be both feasible and scalable^10^. The next generation of clinical prediction models should thus ideally be fielded as an integration with EHR systems.

Despite the promising results, our study has some inherent limitations. As is the case for any retrospective study drawing conclusions from a specific data repository, the results can only be as good as the underlying data. Also, included data points (e.g., AIS and ISS scores) may not be generally available across trauma centers during the initial resuscitation. Furthermore, model performance in actual clinical use will be critically dependent on when input parameters are collected. As such, while all input parameters for the prehospital model were available in the prehospital setting, the temporal nature of these may preclude actual use. As an example of this, parameters such as prehospital cardiac arrest and lowest systolic blood pressure may occur immediately prior to reaching the ED, which would thus alter the model performance significantly compared with what could be achieved at the roadside.

For the TQIP data (as well as other trauma datasets), there is the issue of survivor bias. Indeed, the most severely injured patients suffering prehospital mortality, will never enter the TQIP dataset in the first place.

The combination of survivor bias as well as relatively low trauma complication rates^23^, creates a scenario where the model is faced with predicting rare events, which could skew AUC measurements. For the random forest that predicted early mortality based on the input features from the Pre-Hospital Model, prehospital cardiac arrest was by far the most important, with a relative importance of almost 30%. Examining the data, we found that 52.0% of the patients who died within 24 h had a prehospital cardiac arrest, and that 55.4% of the patients with a prehospital cardiac arrest died within 24 h. Without this input feature, the performance of the random forest algorithm on the test dataset decreased from 0.947 to 0.918.

Input features could also introduce prediction inflation. Indeed, feature importance analyses (Fig. 4) revealed that missing values (e.g., height, weight and systolic blood pressure) ranked highly. Missing values could, however, also indicate a severely injured patient where these measurements could not be obtained due to the urgency of the case, which again underlines why a feature importance analyses should always be assessed in conjunction with a clinical assessment of the presented ROC–AUC values. If we remove all patients with a missing height or weight, the performance on mortality prediction of the random forest decreased further from 0.918 to 0.878.

An additional concern could be the inclusion of early mortality as well as the ED discharge disposition (which includes mortality within 24 h) as inputs for predicting late mortality in the In-Hospital Model. No patients were coded as having experienced both early- and late death. If we removed these input variables, performance decreased from the original test ROC AUC of 0.972 [0.965, 0.978] to 0.951 [0.942, 0.959].

Although the objective of this study is to explore whether moving from static models to models capable of ingesting data is it becomes available, it should be acknowledged that the sliding windows approach using additive data as it becomes available, still has limitations and that true dynamic risk modelling would require time-series models capable of deployment at any given time with the capacity to ingest a heterogenous dataset which may not be identical across patients even at the same point in time, as the dynamic nature of trauma resuscitation will generate a plethora of different data types for each patient at any given time. As such, some patients may have biochemistry information available at time X, others may not but may have radiographic imaging data available etc.

Constructing such models is still an unsolved challenge. We have, however, with this study demonstrated that using additive data as it becomes available for prediction models, could be a potential interesting area for future development in clinical setting with a highly temporal nature of the data.

## Conclusion

Deep neural networks achieve excellent performance in risk stratification of trauma patients as they advance through clinical treatment trajectories.

## Supplementary Information

Supplementary Information.

## Data Availability

The data that support the findings of this study are available from TQIP, but restrictions apply to the availability of these data, which were used under license for the current study, and so are not publicly available. Data are however available from the corresponding authors upon reasonable request and with permission of TQIP. The ACS TQIP database can be requested through the ACS TQIP (https://www.facs.org/quality-programs/trauma/quality/national-trauma-data-bank/datasets/). The dataset is free for institutions participating in the TQIP program and is available on reasonable request to the TQIP steering committee for a service fee for non-participating institutions through the same link.

## Code availability

The code for this study is available at https://github.com/alexbonde/TQIP.
