# Supplementary material for: Assessing the utility of a sliding-windows deep neural network approach for risk prediction of trauma patients
Source: Sci Rep. 2023 Mar 30;13:5176. doi: 10.1038/s41598-023-32453-3 (PMC10063587; doi:10.1038/s41598-023-32453-3)

ONLINE SUPPLEMENTARY MATERIAL

**Supplementary Table 1.** Input- and output variables for all three models along with summary statistics for the training (Train)-, validation (Valid)-, and test (Test) sets.

Categorical data are presented as counts with percentages. Continuous data are presented as medians with interquartile ranges. Missing data is presented were relevant.

EMS: Emergency Medical Services, GCS: Glasgow Coma Scale, ACS: American College of Surgeons, ED: Emergency Department, AIS: Abbreviated Injury Scale, ISS: Injury Severity Score, AQ: Assessment Qualifier, VTE: Venous Thromboembolism, ICU: Intensive Care Unit, LMWH: Low-Molecular-Weight Heparin.

**Table 1A.** Input variables for the Pre-Hospital Model

| **Variable** | **Category** | **Train** | **Valid** | **Test** |
| --- | --- | --- | --- | --- |
| Age | Continuous | 50 [27, 70] | 50 [27, 69] | 34 [15, 60] |
|  | Missing | 43,990 (6.1 %) | 10,915 (6.1 %) | 4,979 (6.1 %) |
| Height | Continuous | 170 [160, 178] | 170 [160, 178] | 168 [155, 178] |
|  | Missing | 102,799 (14.3 %) | 25,715 (14.3 %) | 23,416 (28.9 %) |
| Weight | Continuous | 75 [61, 90] | 75 [61, 90] | 68 [46, 84] |
|  | Missing | 49,008 (6.8 %) | 12,161 (6.8 %) | 9,297 (11.5 %) |
| Gender | Male | 429,608 (59.8 %) | 107,162 (59.7 %) | 51,070 (63.0 %) |
|  | Female | 288,670 (40.2 %) | 72,391 (40.3 %) | 29,967 (37.0 %) |
|  | Missing | 97 (0.0 %) | 40 (0.0 %) | 3 (0.0 %) |
| Race Category: American Indian | No | 711,707 (99.1%) | 177,954 (99.1%) | 80,510 (99.3%) |
|  | Yes | 6,668 (0.9%) | 1,639 (0.9%) | 530 (0.7%) |
| Race Category: Asian | No | 704,839 (98.1%) | 176,116 (98.1%) | 79,674 (98.3%) |
|  | Yes | 13,536 (1.9%) | 3,477 (1.9%) | 1,366 (1.7%) |
| Race Category: Black | No | 620,005 (86.3%) | 154,976 (86.3%) | 64,280 (79.3%) |
|  | Yes | 98,370 (13.7%) | 24,617 (13.7%) | 16,760 (20.7%) |
| Race Category: Other | No | 662,870 (92.3%) | 165,731 (92.3%) | 71,588 (88.3%) |
|  | Yes | 55,505 (7.7%) | 13,862 (7.7%) | 9,452 (11.7%) |
| Race Category: Pacific Islander | No | 716,408 (99.7%) | 179,101 (99.7%) | 80,919 (99.9%) |
|  | Yes | 1,967 (0.3%) | 492 (0.3%) | 121 (0.1%) |
| Race Category: White | No | 187,801 (26.1%) | 47,081 (26.2%) | 29,159 (36.0%) |
|  | Yes | 530,574 (73.9%) | 132,512 (73.8%) | 51,881 (64.0%) |
| Ethnicity | Not Hispanic or Latino | 607,493 (84.6 %) | 151,810 (84.5 %) | 63,161 (77.9 %) |
|  | Hispanic or Latino | 77,976 (10.9 %) | 19,408 (10.8 %) | 12,296 (15.2 %) |
|  | Missing | 32,906 (4.6 %) | 8,375 (4.7 %) | 5,583 (6.9 %) |
| Transport Mode | Ground Ambulance | 540,409 (75.2 %) | 134,762 (75.0 %) | 59,004 (72.8 %) |
|  | Private/ Public Vehicle/ Walk-in | 110,990 (15.5 %) | 27,921 (15.5 %) | 15,120 (18.7 %) |
|  | Helicopter Ambulance | 54,439 (7.6 %) | 13,637 (7.6 %) | 5,373 (6.6 %) |
|  | Other | 3,410 (0.5 %) | 863 (0.5 %) | 92 (0.1 %) |
|  | Fixed-wing Ambulance | 2,982 (0.4 %) | 834 (0.5 %) | 222 (0.3 %) |
|  | Police | 2,358 (0.3 %) | 569 (0.3 %) | 360 (0.4 %) |
|  | Missing | 3,787 (0.5 %) | 1,007 (0.6 %) | 869 (1.1 %) |
| Initial EMS Systolic Blood Pressure | Continuous | 138 [120, 156] | 138 [121, 156] | 136 [119, 153] |
|  | Missing | 324,404 (45.2 %) | 81,583 (45.4 %) | 39,844 (49.2 %) |
| Initial EMS Pulse Rate | Continuous | 88 [76, 102] | 88 [76, 102] | 90 [ 78, 106] |
|  | Missing | 317,477 (44.2 %) | 79,853 (44.5 %) | 38,913 (48.0 %) |
| Initial EMS Respiratory Rate | Continuous | 18 [16, 20] | 18 [16, 20] | 18 [16, 20] |
|  | Missing | 329,167 (45.8 %) | 82,790 (46.1 %) | 40,404 (49.9 %) |
| Initial EMS Oxygen Saturation | Continuous | 97 [95, 99] | 97 [95, 99] | 98 [ 96, 99] |
|  | Missing | 386,044 (53.7 %) | 96,725 (53.9 %) | 45,792 (56.5 %) |
| Initial EMS Total GCS | Continuous | 15 [14, 15] | 15 [15, 15] | 15 [15, 15] |
|  | Missing | 322875 (44.9 %) | 81,044 (45.1 %) | 38,700 (47.8 %) |
| Prehospital Cardiac  Arrest | No | 684,742 (95.3 %) | 171,038 (95.2 %) | 77,740 (95.9 %) |
|  | Yes | 8,543 (1.2 %) | 2,088 (1.2 %) | 958 (1.2 %) |
|  | Missing | 25,090 (3.5 %) | 6,467 (3.6 %) | 2,342 (2.9 %) |
| EMS GCS - Eye | Opens eyes spontaneously | 349,121 (48.6 %) | 86,840 (48.4 %) | 37,350 (46.1 %) |
|  | No eye movement when assessed | 14,627 (2.0 %) | 3,689 (2.1 %) | 1,488 (1.8 %) |
|  | Opens eyes in response to verbal stimulation | 6,514 (0.9 %) | 1,686 (0.9 %) | 755 (0.9 %) |
|  | Opens eyes in response to painful stimulation | 20,654 (2.9 %) | 5,151 (2.9 %) | 2,126 (2.6 %) |
|  | Missing | 327,459 (45.6 %) | 82,227 (45.8 %) | 39,321 (48.5 %) |
| EMS GCS - Verbal | Oriented | 301,755 (42.0 %) | 75,303 (41.9 %) | 32,821 (40.5 %) |
|  | Confused | 54,297 (7.6 %) | 13,411 (7.5 %) | 5,197 (6.4 %) |
|  | No verbal response | 22,658 (3.2 %) | 5,568 (3.1 %) | 2,329 (2.9 %) |
|  | Incomprehensible sounds | 6,671 (0.9 %) | 1,728 (1.0 %) | 757 (0.9 %) |
|  | Inappropriate words | 5,566 (0.8 %) | 1,353 (0.8 %) | 621 (0.8 %) |
|  | Missing | 327,428 (45.6 %) | 82,230 (45.8 %) | 39,315 (48.5 %) |
| EMS GCS - Motor | Obeys commands | 346,759 (48.3 %) | 86,433 (48.1 %) | 37,140 (45.8 %) |
|  | Localizing pain | 14,392 (2.0 %) | 3,558 (2.0 %) | 1,458 (1.8 %) |
|  | Withdrawal from pain | 8,926 (1.2 %) | 2,221 (1.2 %) | 1,002 (1.2 %) |
|  | Flexion to pain | 2,399 (0.3 %) | 568 (0.3 %) | 231 (0.3 %) |
|  | Extension to pain | 1,134 (0.2 %) | 323 (0.2 %) | 116 (0.1 %) |
|  | No motor response | 17,235 (2.4 %) | 4,236 (2.4 %) | 1,763 (2.2 %) |
|  | Missing | 327,530 (45.6 %) | 82,254 (45.8 %) | 39,330 (48.5 %) |
| TCC: Glasgow Coma Scale of 13 or less | No | 693,422 (96.5%) | 173,347 (96.5%) | 78,958 (97.4%) |
|  | Yes | 24,953 (3.5%) | 6,246 (3.5%) | 2,082 (2.6%) |
| TCC: Systolic Blood Pressure under 90 | No | 712,580 (99.2%) | 178,084 (99.2%) | 80,356 (99.2%) |
|  | Yes | 5,795 (0.8%) | 1,509 (0.8%) | 684 (0.8%) |
| TCC: Respiratory rate less than 10 or more than 29 | No | 713,393 (99.3%) | 178,355 (99.3%) | 80,526 (99.4%) |
|  | Yes | 4,982 (0.7%) | 1,238 (0.7%) | 514 (0.6%) |
| TCC: Penetrating Injuries | No | 699,835 (97.4%) | 174,987 (97.4%) | 79,308 (97.9%) |
|  | Yes | 18,540 (2.6%) | 4,606 (2.6%) | 1,732 (2.1%) |
| TCC: Chest wall instability or deformity | No | 717,248 (99.8%) | 179,309 (99.8%) | 80,957 (99.9%) |
|  | Yes | 1,127 (0.2%) | 284 (0.2%) | 83 (0.1%) |
| TCC: Two or more proximal long-bone fractures | No | 715,029 (99.5%) | 178,760 (99.5%) | 80,781 (99.7%) |
|  | Yes | 3,346 (0.5%) | 833 (0.5%) | 259 (0.3%) |
| TCC: Crushed, degloved, mangled, or pulseless extremity | No | 716,127 (99.7%) | 179,019 (99.7%) | 80,923 (99.9%) |
|  | Yes | 2,248 (0.3%) | 574 (0.3%) | 117 (0.1%) |
| TCC: Amputation proximal to wrist or ankle | No | 717,882 (99.9%) | 179,487 (99.9%) | 81,010 (100.0%) |
|  | Yes | 493 (0.1%) | 106 (0.1%) | 30 (0.0%) |
| TCC: Pelvic fracture | No | 715,689 (99.6%) | 178,931 (99.6%) | 80,856 (99.8%) |
|  | Yes | 2,686 (0.4%) | 662 (0.4%) | 184 (0.2%) |
| TCC: Open or depressed skull fracture | No | 716,964 (99.8%) | 179,213 (99.8%) | 80,904 (99.8%) |
|  | Yes | 1,411 (0.2%) | 380 (0.2%) | 136 (0.2%) |
| TCC: Paralysis | No | 716,885 (99.8%) | 179,199 (99.8%) | 80,922 (99.9%) |
|  | Yes | 1,490 (0.2%) | 394 (0.2%) | 118 (0.1%) |
| Hospital Teaching Status | Community | 297,584 (41.4 %) | 74,361 (41.4 %) | 0 (0 %) |
|  | Non-teaching | 130,304 (18.1 %) | 32,589 (18.1 %) | 0 (0 %) |
|  | University | 287,157 (4.0 %) | 71,815 (4.0 %) | 81,040 (1.0 %) |
|  | Missing | 3,330 (0.5 %) | 828 (0.5 %) | 0 (0 %) |
| Bed Size | 200 or less | 73,484 (10.2 %) | 18,370 (10.2 %) | 0 (0 %) |
|  | 201-400 | 191,475 (26.7 %) | 47,923 (26.7 %) | 81,040 (1.0 %) |
|  | 401-600 | 208,223 (29.0 %) | 52,378 (29.2 %) | 0 (0 %) |
|  | More than 600 | 245,193 (34.1 %) | 60,922 (33.9 %) | 0 (0 %) |
|  | Missing | 0 (0 %) | 0 (0 %) | 0 (0 %) |
| Hospital Type | Non-profit | 627,809 (87.4 %) | 157,064 (87.5 %) | 70,461 (86.9 %) |
|  | For Profit | 86,110 (12.0 %) | 21,395 (11.9 %) | 10,579 (13.1 %) |
|  | Government | 1,126 (0.2 %) | 306 (0.2 %) | 0 (0 %) |
|  | Missing | 3,330 (0.5 %) | 828 (0.5 %) | 0 (0 %) |
| ACS Verification Level | Level I | 294,797 (41.0 %) | 73,619 (41.0 %) | 28,552 (35.2 %) |
|  | Level II | 165,420 (23.0 %) | 41,323 (23.0 %) | 10,426 (12.9 %) |
|  | Level III | 46,196 (6.4 %) | 11,571 (6.4 %) | 4,258 (5.3 %) |
|  | Missing | 211,962 (29.5 %) | 53,080 (29.6 %) | 37,804 (46.6 %) |
| Pediatric Verification Level | Level I | 82,254 (11.5 %) | 20,433 (11.4 %) | 18,741 (23.1 %) |
|  | Level II | 76,713 (10.7 %) | 19,269 (10.7 %) | 1,568 (1.9 %) |
|  | Missing | 559,408 (77.9 %) | 139,891 (77.9 %) | 60,731 (74.9 %) |
| State Designation | I | 305,478 (42.5 %) | 76,433 (42.6 %) | 22,999 (28.4 %) |
|  | II | 189,702 (26.4 %) | 47,255 (26.3 %) | 16,247 (20.0 %) |
|  | III | 57,227 (8.0 %) | 14,442 (8.0 %) | 5,161 (6.4 %) |
|  | IV | 1,310 (0.2 %) | 318 (0.2 %) | 0 (0 %) |
|  | Other | 899 (0.1 %) | 225 (0.1 %) | 0 (0 %) |
|  | Missing | 163,759 (22.8 %) | 40,920 (22.8 %) | 36,633 (45.2 %) |
| State Pediatric Designation | I | 78,309 (10.9 %) | 19,476 (10.8 %) | 17,737 (21.9 %) |
|  | II | 40,511 (5.6 %) | 10,314 (5.7 %) | 638 (0.8 %) |
|  | III | 2,765 (0.4 %) | 738 (0.4 %) | 0 (0 %) |
|  | IV | 792 (0.1 %) | 205 (0.1 %) | 0 (0 %) |
|  | Other | 5,497 (0.8 %) | 1,388 (0.8 %) | 0 (0 %) |
|  | Not Applicable | 59,585 (8.3 %) | 14,850 (8.3 %) | 10,980 (13.5 %) |
|  | Missing | 530,916 (73.9 %) | 132,622 (73.8 %) | 51,685 (63.8 %) |
| Primary External Cause | 2,042 values | 1,885 values | 1,318 values | 1,061 values |
|  | Missing | 1044 (0.1 %) | 276 (0.2 %) | 102 (0.1 %) |

**Table 1B.** Additional input variables for the ED Model

| Head Injury* | No | 464,723 (64.7 %) | 116,268 (64.7 %) | 51,919 (64.1 %) |
| --- | --- | --- | --- | --- |
|  | Yes | 253,652 (35.3 %) | 63,325 (35.3 %) | 29,121 (35.9 %) |
| Face Injury* | No | 533,559 (74.3 %) | 133,540 (74.4 %) | 59,741 (73.7 %) |
|  | Yes | 184,816 (25.7 %) | 46,053 (25.6 %) | 21,299 (26.3 %) |
| Neck Injury* | No | 698,239 (97.2 %) | 174,594 (97.2 %) | 78,662 (97.1%) |
|  | Yes | 20,136 (2.8 %) | 4,999 (2.8 %) | 2,378 (2.9%) |
| Thoracic Injury* | No | 544,243 (75.8 %) | 135,965 (75.7 %) | 64,821 (80.0 %) |
|  | Yes | 174,132 (24.2 %) | 43,628 (24.3 %) | 16,219 (20.0 %) |
| Abdominal Injury* | No | 628,579 (87.5 %) | 157,417 (87.7 %) | 71,004 (87.6 %) |
|  | Yes | 89,796 (12.5 %) | 22,176 (12.3 %) | 10,036 (12.4 %) |
| Spine Injury* | No | 596,058 (83.0 %) | 148,882 (82.9 %) | 70,142 (86.6 %) |
|  | Yes | 122,317 (17.0 %) | 30,711 (17.1 %) | 10,898 (13.4 %) |
| Upper Extremity  Injury* | No | 477,082 (66.4 %) | 119,290 (66.4 %) | 53,378 (65.9 %) |
|  | Yes | 241,293 (33.6 %) | 60,303 (33.6 %) | 27,662 (34.1 %) |
| Lower Extremity Injury* | No | 409,575 (57.0 %) | 102,419 (57.0 %) | 50,066 (61.8 %) |
|  | Yes | 308,800 (43.0 %) | 77,174 (43.0 %) | 30,974 (38.2 %) |
| Unspecified Injury* | No | 673,385 (93.7 %) | 168,316 (93.7 %) | 74,658 (92.1 %) |
|  | Yes | 44,990 (6.3 %) | 1,127 (6.3 %) | 6,382 (7.9 %) |
| Time to EMS Response (mins) | Continuous | 8 [0, 14] | 8 [5 ,14] | 8 [5, 14] |
|  | Missing | 255,163 (35.5 %) | 63,810 (35.5 %) | 29,766 (36.7 %) |
| Time EMS spent at scene (mins) | Continuous | 16 [11, 23] | 16 [11, 23] | 16 [11, 23] |
|  | Missing | 250,686 (34.9 %) | 62,722 (34.9 %) | 30,138 (37.2 %) |
| Time from dispatch to ED/hospital arrival (mins) | Continuous | 49 [35, 71] | 49 [35, 71] | 49 [35, 74] |
|  | Missing | 249,122 (34.7 %) | 62,320 (34.7 %) | 29,333 (36.2 %) |
| Initial ED Systolic Blood Pressure | Continuous | 137 [121, 154] | 137 [121, 154] | 131 [117, 149] |
|  | Missing | 23,814 (3.3 %) | 5,966 (3.3 %) | 3,376 (4.2 %) |
| Initial ED Pulse Rate | Continuous | 86 [74, 100] | 87 [74, 100] | 90 [ 77, 106] |
|  | Missing | 16,610 (2.3 %) | 4,113 (2.3 %) | 1,570 (1.9 %) |
| Initial ED Temperature | Continuous | 36.7  [36.4, 36.9] | 36.7  [36.4,36.9] | 36.7  [36.5, 37.0] |
|  | Missing | 73,549 (10.2 %) | 18,449 (10.3 %) | 7,463 (9.2 %) |
| Initial ED Respiratory Rate | Continuous | 18 [16, 20] | 18 [16, 20] | 18 [16, 22] |
|  | Missing | 25,312 (3.5 %) | 6,371 (3.5 %) | 2,563 (3.2 %) |
| Initial ED Oxygen Saturation | Continuous | 98 [96, 99] | 98 [96, 99] | 98 [96, 100] |
|  | Missing | 29,271 (4.1 %) | 7,328 (4.1 %) | 6,809 (8.4 %) |
| AIS derived ISS | Continuous | 9 [4, 10] | 9 [4, 10] | 5 [ 4, 10] |
|  | Missing | 2,396 (0.3 %) | 583 (0.3 %) | 225 (0.3 %) |
| EMS GCS - Eye | Opens eyes spontaneously | 620,345 (86.4 %) | 155,278 (86.5 %) | 70,917 (87.5 %) |
|  | Opens eyes in response to verbal stimulation | 16,121 (2.2 %) | 3,929 (2.2 %) | 1,680 (2.1 %) |
|  | Opens eyes in response to painful stimulation | 5,795 (0.8 %) | 1,437 (0.8 %) | 686 (0.8 %) |
|  | No eye movement when assessed | 35,524 (4.9 %) | 8,855 (4.9 %) | 3,611 (4.5 %) |
|  | Missing | 40,590 (5.7 %) | 10,094 (5.6 %) | 4,146 (5.1 %) |
| EMS GCS - Verbal | Oriented | 567,325 (79.0 %) | 141,934 (79.0 %) | 65,177 (80.4 %) |
|  | Confused | 59,496 (8.3 %) | 14,873 (8.3 %) | 6,447 (8.0 %) |
|  | Inappropriate words | 4,892 (0.7 %) | 1,208 (0.7 %) | 537 (0.7 %) |
|  | Incomprehensible sounds | 6,620 (0.9 %) | 1,689 (0.9 %) | 807 (1.0 %) |
|  | No verbal response | 39,188 (5.5 %) | 9,725 (5.4 %) | 3,896 (4.8 %) |
|  | Missing | 40,854 (5.7 %) | 10,164 (5.7 %) | 4,176 (5.2 %) |
| EMS GCS - Motor | Obeys commands | 622,061 (86.6 %) | 155,586 (86.6 %) | 71,033 (87.7 %) |
|  | No motor response | 29,426 (4.1 %) | 7,275 (4.1 %) | 2,841 (3.5 %) |
|  | Localizing pain | 15,242 (2.1 %) | 3,847 (2.1 %) | 1,835 (2.3 %) |
|  | Withdrawal from pain | 7,643 (1.1 %) | 1,932 (1.1 %) | 827 (1.0 %) |
|  | Flexion to pain | 1,751 (0.2 %) | 421 (0.2 %) | 222 (0.3 %) |
|  | Extension to pain | 1,278 (0.2 %) | 350 (0.2 %) | 137 (0.2 %) |
|  | Missing | 40,974 (5.7) | 10,182 (5.7 %) | 4,145 (5.1 %) |
| GCS AQ - Patient Chemically Sedated or Paralyzed | No | 695,085 (96.8 %) | 173,822 (96.8 %) | 77,790 (96.0 %) |
|  | Yes | 23,290 (3.2 %) | 5,771 (3.2 %) | 3,250 (4.0 %) |
| GCS AQ - Obstruction to the Patient's Eye | No | 715,473 (99.6 %) | 178,889 (99.6 %) | 80,790 (99.7 %) |
|  | Yes | 2,902 (3.6 %) | 704 (0.4 %) | 250 (0.3 %) |
| GCS AQ -  Patient Intubated | No | 692,853 (96.4 %) | 173,216 (96.4 %) | 77,015 (95.0 %) |
|  | Yes | 25,522 (3.6 %) | 6,377 (3.6 %) | 4,025 (5.0 %) |
| GCS AQ -  Valid GCS | No | 57,156 (8.0 %) | 14,196 (7.9 %) | 5,914 (7.3 %) |
|  | Yes | 661,219 (92.0 %) | 165,397 (92.1 %) | 75,126 (98.3 %) |
| Attention Deficit Disorder | No | 709,754 (98.8 %) | 177,479 (98.8 %) | 79,651 (98.3 %) |
|  | Yes | 8,621 (1.2 %) | 21,14 (1.2 %) | 1,389 (1.7 %) |
| Alcohol Use Disorder | No | 680,333 (94.7 %) | 170,108 (94.7 %) | 77,196 (95.3 %) |
|  | Yes | 38,042 (5.3 %) | 9,485 (5.3 %) | 3,844 (4.7 %) |
| Angina Pectoris | No | 717,580 (99.9 %) | 179,413 (99.9 %) | 80,973 (99.9 %) |
|  | Yes | 795 (0.1 %) | 180 (0.1 %) | 67 (0.1 %) |
| Anticoagulant Therapy | No | 663,691 (92.4 %) | 166,112 (92.5 %) | 76,845 (94.8 %) |
|  | Yes | 54,684 (7.6 %) | 13,481 (7.5 %) | 4,195 (5.2 %) |
| Bleeding Disorder | No | 707,232 (98.4 %) | 176,820 (98.5 %) | 79,881 (98.6 %) |
|  | Yes | 11,143 (1.6 %) | 2,773 (1.5 %) | 1,159 (1.4 %) |
| Currently Receiving Chemotherapy for Cancer | No | 715,803 (99.6 %) | 178,957 (99.6 %) | 80,809 (99.7 %) |
|  | Yes | 2,572 (0.4 %) | 636 (0.4 %) | 231 (0.3 %) |
| Cirrhosis | No | 711,231 (99.0 %) | 177,821 (99.0 %) | 80,485 (99.3 %) |
|  | Yes | 7,144 (1.0 %) | 1,772 (1.0 %) | 555 (0.7 %) |
| Chronic Obstructive Pulmonary Disease | No | 673,113 (93.7 %) | 168,305 (93.7 %) | 77,321 (95.4 %) |
|  | Yes | 45,262 (6.3 %) | 11,288 (6.3 %) | 3,719 (4.6 %) |
| Cerebrovascular Accident | No | 69,9713 (97.4 %) | 174,966 (97.4 %) | 79,540 (98.1 %) |
|  | Yes | 18,662 (2.6 %) | 4,627 (2.6 %) | 1,500 (1.9 %) |
| Dementia | No | 679,742 (94.6 %) | 169,992 (94.7 %) | 78,276 (96.6 %) |
|  | Yes | 38,633 (5.4 %) | 9,601 (5.3 %) | 2,764 (3.4 %) |
| Diabetes Mellitus | No | 628,272 (87.5 %) | 157,087 (87.5 %) | 73,504 (90.7 %) |
|  | Yes | 90,103 (12.5 %) | 22,506 (12.5 %) | 7,536 (9.3 %) |
| Disseminated Cancer | No | 714,309 (99.4 %) | 178,606 (99.5 %) | 80,768 (99.7 %) |
|  | Yes | 4,066 (0.6 %) | 987 (0.5 %) | 272 (0.3 %) |
| Functionally Dependent Health Status | No | 666,709 (92.8 %) | 166,788 (92.9 %) | 77,107 (95.1 %) |
|  | Yes | 51,666 (7.2 %) | 12,805 (7.1 %) | 3,933 (4.9 %) |
| Congestive Heart Failure | No | 690,825 (96.2 %) | 172,628 (96.1 %) | 79,125 (97.6 %) |
|  | Yes | 27,550 (3.8 %) | 6,965 (3.9 %) | 1,915 (2.4 %) |
| Hypertension | No | 488,891 (68.1 %) | 122,076 (68.0 %) | 62,975 (77.7 %) |
|  | Yes | 229,484 (31.9 %) | 57,517 (32.0 %) | 18,065 (22.3 %) |
| Myocardial Infarction | No | 711,935 (99.1 %) | 177,966 (99.1 %) | 80,580 (99.4 %) |
|  | Yes | 6,440 (0.9 %) | 1,627 (0.9 %) | 460 (0.6 %) |
| Peripheral Arterial Disease | No | 714,700 (99.5 %) | 178,642 (99.5 %) | 80,755 (99.6 %) |
|  | Yes | 3,675 (0.5 %) | 951 (0.5 %) | 285 (0.4 %) |
| Other Comorbidity | No | 595,228 (82.9 %) | 148,910 (82.9 %) | 69,463 (85.7 %) |
|  | Yes | 123,147 (17.1 %) | 30,683 (17.1 %) | 11,577 (14.3 %) |
| Mental/Personality Disorder | No | 649,566 (90.4 %) | 162,719 (90.6 %) | 74,710 (92.2 %) |
|  | Yes | 68,809 (9.6 %) | 16,874 (9.4 %) | 6,330 (7.8 %) |
| Chronic Renal Failure | No | 707,199 (98.4 %) | 176,713 (98.4 %) | 80,125 (98.9 %) |
|  | Yes | 11,176 (1.6 %) | 2,880 (1.6 %) | 915 (1.1 %) |
| Current Smoker | No | 587,590 (81.8 %) | 147,024 (81.9 %) | 69,697 (86.0 %) |
|  | Yes | 130,785 (18.2 %) | 32,569 (18.1 %) | 11,343 (14.0 %) |
| Steroid Use | No | 712,568 (99.2 %) | 178,174 (99.2 %) | 80,594 (99.4 %) |
|  | Yes | 5,807 (0.8 %) | 1,419 (0.8 %) | 446 (0.6 %) |
| Substance Abuse Disorder | No | 681,740 (94.9 %) | 170,379 (94.9 %) | 76,836 (94.8 %) |
|  | Yes | 36,635 (5.1 %) | 9,214 (5.1 %) | 4,204 (5.2 %) |
| Respiratory Assistance | No | 652,326 (90.8 %) | 162,958 (90.7 %) | 75,848 (93.6 %) |
|  | Yes | 35,586 (5.0 %) | 8,853 (4.9 %) | 3,125 (3.9 %) |
|  | Missing | 30,463 (4.2 %) | 7,782 (4.3 %) | 2,067 (2.6 %) |
| Supplemental Oxygen | No | 559,270 (77.9 %) | 139,960 (77.9 %) | 62,762 (77.4 %) |
|  | Yes | 109,710 (15.3 %) | 27,323 (15.2 %) | 11,031 (13.6 %) |
|  | Missing | 49,395 (6.9 %) | 12,310 (6.9 %) | 7,247 (8.9 %) |

**We chose to one-hot encode the severity of each injured body region. As one patient can have multiple injuries of different severities in the same- or other body regions, we have simplified the summary statistic to display a binary yes/no value.*

**Table 1C.** Additional input variables for the In-Hospital Model

| VTE Prophylaxis – If administrated within the first day of hospitalization | None | 486,845 (67.8 %) | 121,636 (67.7 %) | 53,931 (66.5 %) |
| --- | --- | --- | --- | --- |
|  | LMWH (Dalteparin, Enoxaparin, etc.) | 105,901 (14.7 %) | 26,512 (14.8 %) | 6,894 (8.5 %) |
|  | Heparin | 37,631 (5.2 %) | 9,403 (5.2 %) | 3,598 (4.4 %) |
|  | Other | 4,239 (0.6 %) | 1,073 (0.6 %) | 309 (0.4 %) |
|  | Xa Inhibitor (Rivaroxaban, etc.) | 1,711 (0.2 %) | 417 (0.2 %) | 92 (0.1 %) |
|  | Coumadin | 1,594 (0.2 %) | 388 (0.2 %) | 110 (0.1 %) |
|  | Direct Thrombin Inhibitor (Dabigatran, etc.) | 315 (0.0 %) | 74 (0.0 %) | 11 (0.0 %) |
|  | Missing | 80,139 (11.2 %) | 20,090 (11.2 %) | 16,095 (19.9 %) |
| Surgery for Hemorrhage Control – If performed within the first day of hospitalization | None | 13,676 (1.9 %) | 3,300 (1.8 %) | 1,352 (1.7 %) |
|  | Laparotomy | 6,607 (0.9 %) | 1,653 (0.9 %) | 647 (0.8 %) |
|  | Thoracotomy | 1,694 (0.2 %) | 407 (0.2 %) | 190 (0.2 %) |
|  | Extremity | 1,549 (0.2 %) | 370 (0.2 %) | 147 (0.2 %) |
|  | Other skin/soft tissue | 614 (0.1 %) | 177 (0.1 %) | 66 (0.1 %) |
|  | Mangled extremity/traumatic amputation | 572 (0.1 %) | 121 (0.1 %) | 36 (0.0 %) |
|  | Neck | 333 (0.0 %) | 71 (0.0 %) | 44 (0.1 %) |
|  | Sternotomy | 197 (0.0 %) | 46 (0.0 %) | 25 (0.0 %) |
|  | Missing | 693,133 (96.5 %) | 173,448 (96.6 %) | 78,533 (96.9 %) |
| Discharge Disposition – If discharged within the first day of hospitalization | Floor bed (general admission, non-specialty unit bed | 301,501 (42.0 %) | 75,093 (41.8 %) | 32,770 (40.4 %) |
|  | Home without services | 60,565 (8.4 %) | 15,094 (8.4 %) | 10,840 (13.4 %) |
|  | Intensive Care Unit (ICU) | 133,835 (18.6 %) | 33,602 (18.7 %) | 13,130 (16.2 %) |
|  | Observation unit (unit that provides < 24 hour stays) | 22,258 (3.1 %) | 5,642 (3.1 %) | 5,758 (7.1 %) |
|  | Operating Room | 78,537 (10.9 %) | 19,524 (10.9 %) | 7,951 (9.8 %) |
|  | Telemetry/step-down unit (less acuity than ICU) | 59,337 (8.3 %) | 14,946 (8.3 %) | 4,461 (5.5 %) |
|  | Transferred to another hospital | 27,151 (3.8 %) | 6,762 (3.8 %) | 2,096 (2.6 %) |
|  | Deceased | 6,624 (0.9 %) | 1,671 (0.9 %) | 644 (0.8 %) |
|  | None | 2,992 (0.4 %) | 841 (0.5 %) | 483 (0.6 %) |
|  | Other (jail, institutional care, mental health, etc.) | 2,276 (0.3 %) | 574 (0.3 %) | 240 (0.3 %) |
|  | Left against medical advice | 1,586 (0.2 %) | 424 (0.2 %) | 271 (0.3 %) |
|  | Home with services | 1,376 (0.2 %) | 310 (0.2 %) | 79 (0.1 %) |
|  | Missing | 20,337 (2.8 %) | 5,110 (2.8 %) | 2,317 (2.9 %) |
| Deceased – If deceased within the first day of hospitalization | No | 709,124 (98.7 %) | 177,308 (98.7 %) | 80,173 (98.9 %) |
|  | Yes | 9,251 (1.3 %) | 2,285 (1.3 %) | 867 (1.1 %) |

**Table 1D.** Output variables. Values indicate number of patients (percentage of the total dataset).

| Central Line-Associated Bloodstream Infection | No | 718,099 (100 %) | 179,528 (100 %) | 81,023 (100 %) |
| --- | --- | --- | --- | --- |
|  | Yes | 276 (0.00 %) | 65 (0.00 %) | 17 (0.00 %) |
| Deep Surgical Site Infection | No | 717,727 (99.9 %) | 179,428 (99.9 %) | 80,991 (99.9 %) |
|  | Yes | 648 (0.1 %) | 165 (0.1 %) | 49 (0.1 %) |
| Deep Vein Thrombosis | No | 714,893 (99.5 %) | 178,769 (99.5 %) | 80,686 (99.6 %) |
|  | Yes | 3,482 (0.5 %) | 824 (0.5 %) | 354 (0.4 %) |
| Cardiac Arrest | No | 714,248 (99.4 %) | 178,553 (99.4 %) | 80,695 (99.6 %) |
|  | Yes | 4,127 (0.6 %) | 1,040 (0.6 %) | 345 (0.4 %) |
| Catheter-Associated Urinary Tract Infection | No | 716,881 (99.8 %) | 179,227 (99.8 %) | 80,932 (99.9 %) |
|  | Yes | 1,494 (0.2 %) | 366 (0.2 %) | 108 (0.1 %) |
| Pulmonary Embolism | No | 716,639 (99.8 %) | 179,157 (99.8 %) | 80,865 (99.8 %) |
|  | Yes | 1,736 (0.2 %) | 436 (0.2 %) | 175 (0.2 %) |
| Extremity Compartment Syndrome | No | 717,803 (99.9 %) | 179,447 (99.9 %) | 80,983 (99.9 %) |
|  | Yes | 572 (0.1 %) | 146 (0.1 %) | 57 (0.1 %) |
| Unplanned Intubation | No | 712,683 (99.2 %) | 178,172 (99.2 %) | 80,539 (99.4 %) |
|  | Yes | 5,692 (0.8 %) | 1,421 (0.8 %) | 501 (0.6 %) |
| Acute Kidney Injury | No | 715,191 (99.6 %) | 178,810 (99.6 %) | 80,780 (99.7 %) |
|  | Yes | 3,184 (0.4 %) | 783 (0.4 %) | 260 (0.3 %) |
| Myocardial Infarction | No | 717,323 (99.9 %) | 179,317 (99.8 %) | 80,967 (99.9 %) |
|  | Yes | 1,052 (0.1 %) | 276 (0.2 %) | 73 (0.1 %) |
| Organ/Space Surgical Site Infection | No | 717,951 (99.9 %) | 179,456 (99.9 %) | 80,996 (99.9 %) |
|  | Yes | 424 (0.1 %) | 137 (0.1 %) | 44 (0.1 %) |
| Acute Respiratory Distress Syndrome | No | 716,480 (99.7 %) | 179,165 (99.8 %) | 80,873 (99.8 %) |
|  | Yes | 1,895 (0.3 %) | 428 (0.2 %) | 167 (0.2 %) |
| Severe Sepsis | No | 716,563 (99.7 %) | 179,158 (99.8 %) | 80,893 (99.8 %) |
|  | Yes | 1,812 (0.3 %) | 435 (0.2 %) | 147 (0.2 %) |
| Stroke / Cerebrovascular Accident | No | 716,873 (99.8 %) | 179,265 (99.8 %) | 80,928 (99.9 %) |
|  | Yes | 1,502 (0.2 %) | 328 (0.2 %) | 112 (0.1 %) |
| Superficial Incisional Surgical Site Infection | No | 717,778 (99.9 %) | 179,472 (99.9 %) | 80,995 (99.9 %) |
|  | Yes | 597 (0.1 %) | 121 (0.1 %) | 45 (0.1 %) |
| Pressure Ulcer | No | 716,202 (99.7 %) | 179,083 (99.7 %) | 80,847 (99.8 %) |
|  | Yes | 2,173 (0.3 %) | 510 (0.3 %) | 193 (0.2 %) |
| Ventilator-Associated Pneumonia | No | 715,410 (99.6 %) | 178,885 (99.6 %) | 80,789 (99.7 %) |
|  | Yes | 2,965 (0.4 %) | 708 (0.4 %) | 251 (0.3 %) |
| Mortality - Within the first day of hospitalization * | No | 709,124 (98.7 %) | 177,308 (98.7 %) | 80,173 (98.9 %) |
|  | Yes | 9,251 (1.3 %) | 2,285 (1.3 %) | 867 (1.1 %) |
| Mortality - After the first day of hospitalization | No | 703,366 (97.9 %) | 175,850 (97.9 %) | 79,795 (98.5 %) |
|  | Yes | 15,009 (2.1 %) | 3,743 (2.1 %) | 1,245 (1.5 %) |

*** Mortality – Within the first day of hospitalization, was not an outcome variable for the In-Hospital Model, as this model was intended for use after the first day of hospitalization.

**Supplementary Table 2.** Performance metrics for each of the three models, both on the validation and on the test datasets.

Presented as the area under the receiver characteristics curve with 95% confidence intervals. ED: Emergency Department.

|  | **Pre-Hospital Model** | | **ED**  **Model** | | **In-Hospital Model** | |
| --- | --- | --- | --- | --- | --- | --- |
|  | **Valid** | **Test** | **Valid** | **Test** | **Valid** | **Test** |
| Central Line-Associated Bloodstream Infection | 0.864 [0.811, 0.917] | 0.819 [0.696, 0.941] | 0.948 [0.913, 0.983] | 0.899 [0.801, 0.998] | 0.957 [0.925, 0.989] | 0.904 [0.808, 1.000] |
| Catheter-Associated Urinary Tract Infection | 0.836 [0.810, 0.861] | 0.804 [0.754, 0.854] | 0.917 [0.897, 0.936] | 0.903 [0.865, 0.942] | 0.923 [0.904, 0.942] | 0.911 [0.874, 0.948] |
| Superficial Surgical Site Infection | 0.851 [0.811, 0.891] | 0.825 [0.751, 0.900] | 0.928 [0.899, 0.958] | 0.880 [0.815, 0.945] | 0.932 [0.903, 0.961] | 0.884 [0.820, 0.948] |
| Deep Surgical Site Infection | 0.883 [0.850, 0.915] | 0.821 [0.749, 0.893] | 0.951 [0.929, 0.973] | 0.910 [0.854, 0.965] | 0.958 [0.938, 0.979] | 0.922 [0.870, 0.974] |
| Organ Space Surgical Site Infection | 0.887 [0.847, 0.927] | 0.909 [0.850, 0.967] | 0.951 [0.923, 0.979] | 0.937 [0.887, 0.987] | 0.951 [0.923, 0.978] | 0.943 [0.895, 0.991] |
| Ventilator-Associated Pneumonia | 0.912 [0.897, 0.926] | 0.864 [0.835, 0.892] | 0.970 [0.961, 0.979] | 0.960 [0.943, 0.977] | 0.973 [0.964, 0.981] | 0.967 [0.951, 0.982] |
| Severe Sepsis | 0.830 [0.807, 0.852] | 0.804 [0.761, 0.846] | 0.924 [0.908, 0.941] | 0.912 [0.880, 0.944] | 0.928 [0.912, 0.944] | 0.923 [0.894, 0.953] |
| Deep Vein Thrombosis | 0.816 [0.799, 0.834] | 0.839 [0.813, 0.865] | 0.900 [0.886, 0.914] | 0.907 [0.887, 0.928] | 0.908 [0.895, 0.921] | 0.909 [0.888, 0.929] |
| Pulmonary Embolism | 0.810 [0.786, 0.834] | 0.837 [0.800, 0.874] | 0.899 [0.880, 0.917] | 0.918 [0.890, 0.946] | 0.901 [0.882, 0.919] | 0.914 [0.885, 0.942] |
| Extremity Compartment Syndrome | 0.854 [0.810, 0.897] | 0.779 [0.707, 0.850] | 0.954 [0.928, 0.981] | 0.905 [0.852, 0.957] | 0.951 [0.923, 0.978] | 0.913 [0.862, 0.963] |
| Pressure Ulcer | 0.846 [0.825, 0.867] | 0.804 [0.766, 0.841] | 0.935 [0.920, 0.950] | 0.907 [0.879, 0.936] | 0.936 [0.922, 0.951] | 0.914 [0.887, 0.942] |
| Acute Kidney Injury | 0.823 [0.806, 0.840] | 0.822 [0.791, 0.853] | 0.906 [0.893, 0.920] | 0.889 [0.863, 0.915] | 0.910 [0.897, 0.924] | 0.894 [0.869, 0.920] |
| Myocardial infarction | 0.844 [0.815, 0.872] | 0.846 [0.790, 0.902] | 0.910 [0.887, 0.933] | 0.866 [0.812, 0.919] | 0.916 [0.894, 0.939] | 0.878 [0.826, 0.930] |
| Cardiac Arrest | 0.880 [0.867, 0.893] | 0.893 [0.870, 0.915] | 0.943 [0.934, 0.953] | 0.943 [0.926, 0.960] | 0.949 [0.940, 0.958] | 0.942 [0.922, 0.962] |
| Stroke | 0.835 [0.810, 0.860] | 0.803 [0.754, 0.852] | 0.911 [0.891, 0.930] | 0.882 [0.842, 0.923] | 0.914 [0.895, 0.933] | 0.858 [0.814, 0.902] |
| Unplanned Intubation | 0.803 [0.790, 0.816] | 0.821 [0.799, 0.844] | 0.907 [0.897, 0.917] | 0.908 [0.890, 0.925] | 0.913 [0.903, 0.923] | 0.913 [0.895, 0.930] |
| Acute Respiratory Distress Syndrome | 0.856 [0.835, 0.877] | 0.798 [0.757, 0.838] | 0.941 [0.927, 0.956] | 0.905 [0.874, 0.936] | 0.943 [0.929, 0.957] | 0.912 [0.882, 0.942] |
| **Mean** | **0.849** | **0.829** | **0.929** | **0.908** | **0.933** | **0.912** |
| Mortality - Within the first day of hospitalization | 0.980 [0.976, 0.984] | 0.980 [0.973, 0.986] | 0.994 [0.992, 0.996] | 0.994 [0.991, 0.998] |  |  |
| Mortality - After the first day of hospitalization | 0.891 [0.884, 0.898] | 0.910 [0.899, 0.922] | 0.956 [0.952, 0.961] | 0.966 [0.958, 0.973] | 0.963 [0.959, 0.968] | 0.972 [0.965, 0.978] |

**Supplementary Table 3.** Performance metrics for each of the three models, both on the validation- and on the test sets.

Presented as the Brier Score.

|  | **Pre-Hospital Model** | | **ED**  **Model** | | **In-Hospital Model** | |
| --- | --- | --- | --- | --- | --- | --- |
|  | **Valid** | **Test** | **Valid** | **Test** | **Valid** | **Test** |
| Central Line-Associated Bloodstream Infection | 0.00040 | 0.00021 | 0.00040 | 0.00021 | 0.00040 | 0.00022 |
| Catheter-Associated Urinary Tract Infection | 0.00197 | 0.00133 | 0.00194 | 0.00132 | 0.00194 | 0.00134 |
| Superficial Surgical Site Infection | 0.00076 | 0.00055 | 0.00075 | 0.00055 | 0.00076 | 0.00056 |
| Deep Surgical Site Infection | 0.00095 | 0.00060 | 0.00093 | 0.00060 | 0.00093 | 0.00060 |
| Organ Space Surgical Site Infection | 0.00062 | 0.00054 | 0.00060 | 0.00053 | 0.00060 | 0.00053 |
| Ventilator-Associated Pneumonia | 0.00384 | 0.00305 | 0.00364 | 0.00295 | 0.00365 | 0.00296 |
| Severe Sepsis | 0.00257 | 0.00180 | 0.00251 | 0.00179 | 0.00251 | 0.00180 |
| Deep Vein Thrombosis | 0.00473 | 0.00427 | 0.00464 | 0.00418 | 0.00463 | 0.00422 |
| Pulmonary Embolism | 0.00253 | 0.00214 | 0.00250 | 0.00212 | 0.00250 | 0.00214 |
| Extremity Compartment Syndrome | 0.00063 | 0.00070 | 0.00062 | 0.00070 | 0.00062 | 0.00071 |
| Pressure Ulcer | 0.00279 | 0.00237 | 0.00271 | 0.00234 | 0.00272 | 0.00237 |
| Acute Kidney Injury | 0.00450 | 0.00317 | 0.00434 | 0.00313 | 0.00433 | 0.00314 |
| Myocardial infarction | 0.00154 | 0.00090 | 0.00152 | 0.00090 | 0.00153 | 0.00090 |
| Cardiac Arrest | 0.00577 | 0.00411 | 0.00547 | 0.00401 | 0.00531 | 0.00298 |
| Stroke | 0.00214 | 0.00138 | 0.00211 | 0.00138 | 0.00211 | 0.00140 |
| Unplanned Intubation | 0.00808 | 0.00606 | 0.00779 | 0.00590 | 0.00777 | 0.00595 |
| Acute Respiratory Distress Syndrome | 0.00265 | 0.00205 | 0.00259 | 0.00203 | 0.00259 | 0.00204 |
| **Mean** | **0.00273** | **0.00207** | **0.00265** | **0.00204** | **0.00264** | **0.00199** |
| Mortality - Within the first day of hospitalization | 0.00683 | 0.00497 | 0.00524 | 0.00339 |  |  |
| Mortality - After the first day of hospitalization | 0.01707 | 0.01285 | 0.01459 | 0.01066 | 0.01343 | 0.00968 |

**Supplementary Table 4.** Performance metrics for the random forest algorithms, both on the validation- and on the test sets.

Presented as the area under the receiver characteristics curve with 95% confidence intervals.

|  | **Pre-Hospital Model** | | **ED**  **Model** | | **In-Hospital Model** | |
| --- | --- | --- | --- | --- | --- | --- |
|  | **Valid** | **Test** | **Valid** | **Test** | **Valid** | **Test** |
| Central Line-Associated Bloodstream Infection | 0.640 [0.568, 0.713] | 0.611 [0.468, 0.754] | 0.788 [0.722, 0.854] | 0.667 [0.525, 0.809] | 0.791 [0.726, 0.857] | 0.783 [0.654, 0.913] |
| Catheter-Associated Urinary Tract Infection | 0.717 [0.687, 0.747] | 0.737 [0.683, 0.791] | 0.806 [0.779, 0.833] | 0.835 [0.788, 0.882] | 0.813 [0.786, 0.840] | 0.834 [0.786, 0.881] |
| Superficial Surgical Site Infection | 0.598 [0.544, 0.651] | 0.570 [0.483, 0.657] | 0.696 [0.644, 0.749] | 0.770 [0.689, 0.851] | 0.710 [0.658, 0.762] | 0.736 [0.652, 0.820] |
| Deep Surgical Site Infection | 0.701 [0.656, 0.746] | 0.651 [0.567, 0.735] | 0.801 [0.761, 0.842] | 0.786 [0.709, 0.862] | 0.829 [0.791, 0.868] | 0.819 [0.747, 0.892] |
| Organ Space Surgical Site Infection | 0.724 [0.676, 0.773] | 0.580 [0.491, 0.668] | 0.817 [0.774, 0.860] | 0.824 [0.748, 0.899] | 0.847 [0.806, 0.887] | 0.837 [0.763, 0.911] |
| Ventilator-Associated Pneumonia | 0.826 [0.807, 0.844] | 0.804 [0.771, 0.837] | 0.915 [0.901, 0.929] | 0.946 [0.927, 0.966] | 0.921 [0.907, 0.935] | 0.947 [0.928, 0.966] |
| Severe Sepsis | 0.703 [0.676, 0.731] | 0.671 [0.623, 0.719] | 0.831 [0.807, 0.855] | 0.881 [0.845, 0.917] | 0.827 [0.803, 0.850] | 0.879 [0.843, 0.915] |
| Deep Vein Thrombosis | 0.716 [0.696, 0.736] | 0.731 [0.700, 0.761] | 0.822 [0.805, 0.840] | 0.817 [0.790, 0.844] | 0.827 [0.809, 0.844] | 0.821 [0.795, 0.848] |
| Pulmonary Embolism | 0.631 [0.602, 0.659] | 0.657 [0.613, 0.701] | 0.747 [0.720, 0.773] | 0.839 [0.802, 0.876] | 0.752 [0.725, 0.778] | 0.852 [0.816, 0.888] |
| Extremity Compartment Syndrome | 0.653 [0.605, 0.702] | 0.618 [0.540, 0.696] | 0.767 [0.722, 0.813] | 0.792 [0.722, 0.862] | 0.772 [0.727, 0.817] | 0.855 [0.793, 0.917] |
| Pressure Ulcer | 0.729 [0.704, 0.755] | 0.713 [0.672, 0.754] | 0.832 [0.810, 0.853] | 0.874 [0.842, 0.906] | 0.829 [0.807, 0.851] | 0.876 [0.845, 0.908] |
| Acute Kidney Injury | 0.720 [0.700, 0.740] | 0.789 [0.756, 0.822] | 0.808 [0.790, 0.827] | 0.868 [0.840, 0.896] | 0.816 [0.798, 0.835] | 0.879 [0.852, 0.906] |
| Myocardial infarction | 0.682 [0.647, 0.717] | 0.707 [0.640, 0.774] | 0.747 [0.713, 0.780] | 0.851 [0.795, 0.906] | 0.740 [0.706, 0.773] | 0.808 [0.748, 0.869] |
| Cardiac Arrest | 0.811 [0.795, 0.827] | 0.810 [0.782, 0.837] | 0.889 [0.876, 0.902] | 0.928 [0.909, 0.947] | 0.895 [0.882, 0.908] | 0.917 [0.893, 0.940] |
| Stroke | 0.668 [0.636, 0.700] | 0.735 [0.682, 0.789] | 0.770 [0.740, 0.800] | 0.816 [0.768, 0.864] | 0.791 [0.761, 0.820] | 0.842 [0.796, 0.887] |
| Unplanned Intubation | 0.717 [0.702, 0.732] | 0.738 [0.713, 0.763] | 0.830 [0.817, 0.843] | 0.888 [0.869, 0.907] | 0.839 [0.826, 0.851] | 0.894 [0.876, 0.913] |
| Acute Respiratory Distress Syndrome | 0.730 [0.703, 0.757] | 0.739 [0.696, 0.783] | 0.835 [0.811, 0.859] | 0.871 [0.837, 0.906] | 0.833 [0.810, 0.857] | 0.880 [0.846, 0.913] |
| **Mean** | **0.704** | **0.698** | **0.806** | **0.838** | **0.814** | **0.851** |
| Mortality - Within the first day of hospitalization | 0.962 [0.956, 0.967] | 0.947 [0.937, 0.958] | 0.986 [0.982, 0.989] | 0.992 [0.988, 0.996] |  |  |
| Mortality - After the first day of hospitalization | 0.857 [0.849, 0.865] | 0.867 [0.854, 0.880] | 0.933 [0.928, 0.939] | 0.951 [0.942, 0.959] | 0.939 [0.934, 0.945] | 0.956 [0.948, 0.964] |

**Supplementary Table 5.** Performance metrics for the random forest algorithms, both on the validation- and on the test sets.

Presented as the Brier Score.

|  | **Pre-Hospital Model** | | **ED**  **Model** | | **In-Hospital Model** | |
| --- | --- | --- | --- | --- | --- | --- |
|  | **Valid** | **Test** | **Valid** | **Test** | **Valid** | **Test** |
| Central Line-Associated Bloodstream Infection | 0.00036 | 0.00021 | 0.00036 | 0.00027 | 0.00036 | 0.00025 |
| Catheter-Associated Urinary Tract Infection | 0.00203 | 0.00134 | 0.00203 | 0.00152 | 0.00202 | 0.00154 |
| Superficial Surgical Site Infection | 0.00068 | 0.00056 | 0.00068 | 0.00060 | 0.00068 | 0.00061 |
| Deep Surgical Site Infection | 0.00092 | 0.00061 | 0.00091 | 0.00063 | 0.00091 | 0.00064 |
| Organ Space Surgical Site Infection | 0.00076 | 0.00055 | 0.00075 | 0.00057 | 0.00075 | 0.00057 |
| Ventilator-Associated Pneumonia | 0.00386 | 0.00312 | 0.00377 | 0.00316 | 0.00376 | 0.00320 |
| Severe Sepsis | 0.00242 | 0.00184 | 0.00239 | 0.00192 | 0.00239 | 0.00196 |
| Deep Vein Thrombosis | 0.00455 | 0.00441 | 0.00450 | 0.00502 | 0.00449 | 0.00504 |
| Pulmonary Embolism | 0.00243 | 0.00218 | 0.00242 | 0.00223 | 0.00242 | 0.00225 |
| Extremity Compartment Syndrome | 0.00082 | 0.00072 | 0.00081 | 0.00074 | 0.00081 | 0.00074 |
| Pressure Ulcer | 0.00283 | 0.00241 | 0.00279 | 0.00252 | 0.00279 | 0.00254 |
| Acute Kidney Injury | 0.00433 | 0.00327 | 0.00425 | 0.00333 | 0.00424 | 0.00332 |
| Myocardial infarction | 0.00154 | 0.00092 | 0.00154 | 0.00095 | 0.00153 | 0.00095 |
| Cardiac Arrest | 0.00558 | 0.00429 | 0.00544 | 0.00422 | 0.00522 | 0.00314 |
| Stroke | 0.00183 | 0.00141 | 0.00182 | 0.00146 | 0.00182 | 0.00147 |
| Unplanned Intubation | 0.00784 | 0.00622 | 0.00770 | 0.00626 | 0.00768 | 0.00628 |
| Acute Respiratory Distress Syndrome | 0.00238 | 0.00209 | 0.00235 | 0.00218 | 0.00235 | 0.00215 |
| **Mean** | **0.00266** | **0.00213** | **0.00262** | **0.00221** | **0.00260** | **0.00216** |
| Mortality - Within the first day of hospitalization | 0.00662 | 0.00613 | 0.00506 | 0.00398 |  |  |
| Mortality - After the first day of hospitalization | 0.01775 | 0.01368 | 0.01542 | 0.01180 | 0.01418 | 0.01092 |

**Supplementary Figure 1A.** Random Forest Feature Importance – Pre-Hospital Model


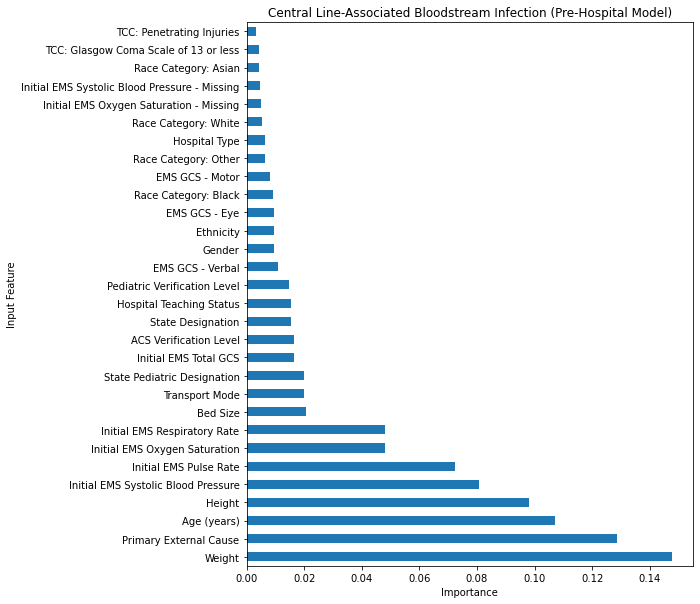

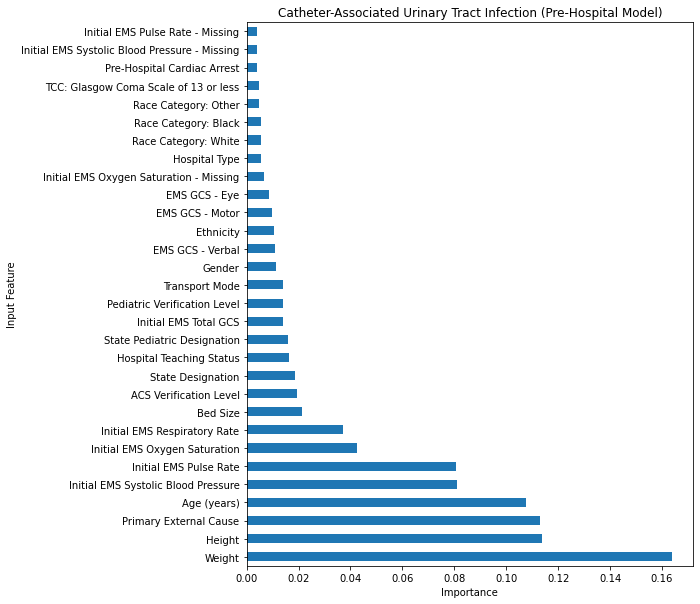

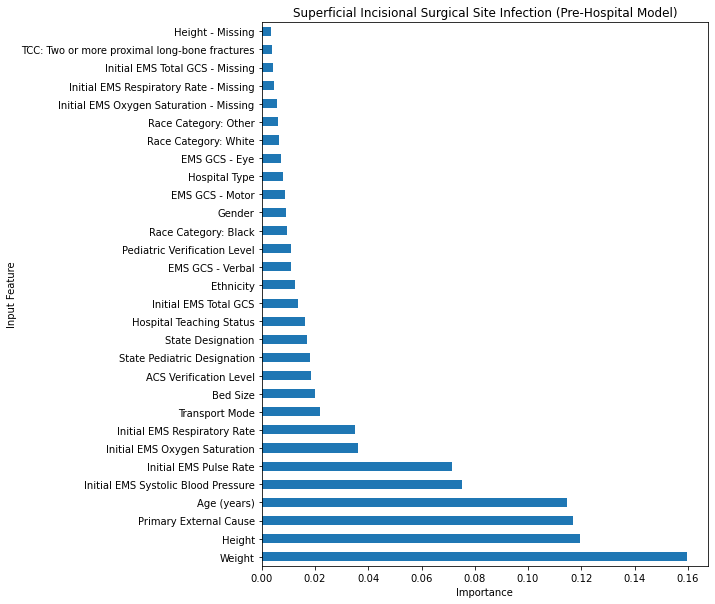

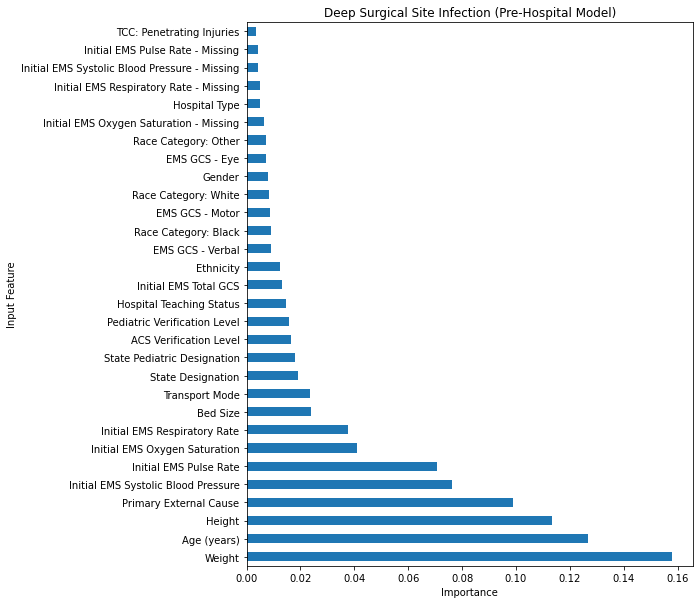

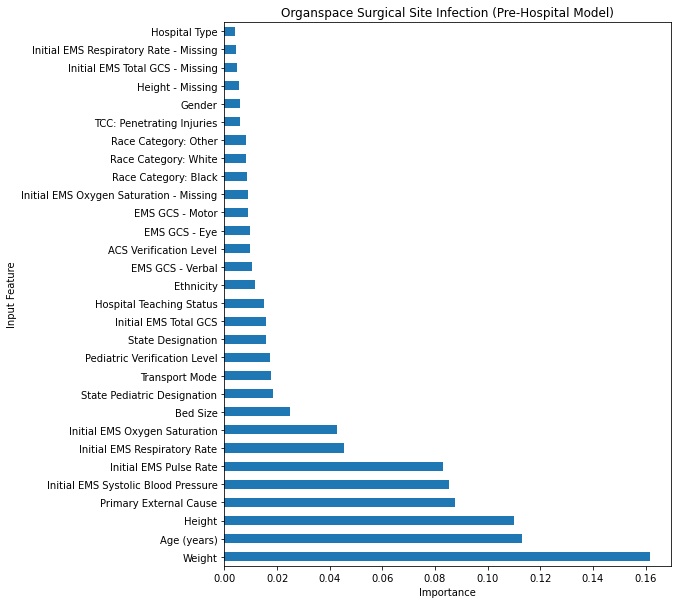

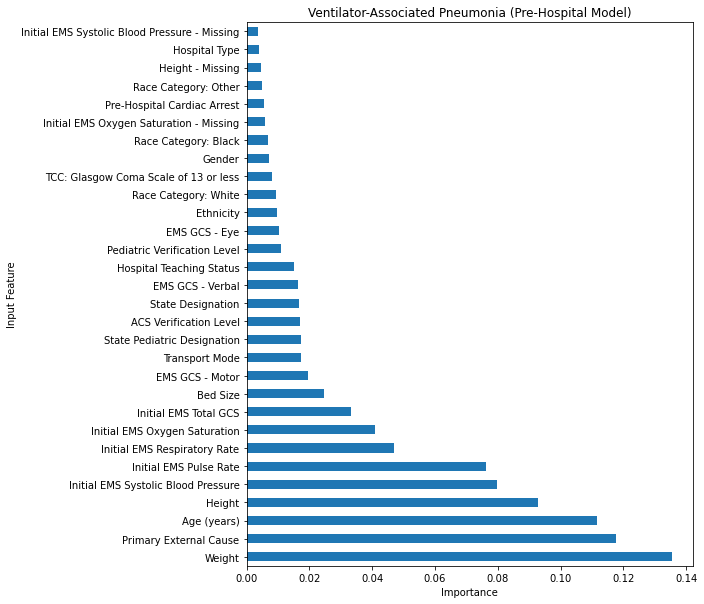


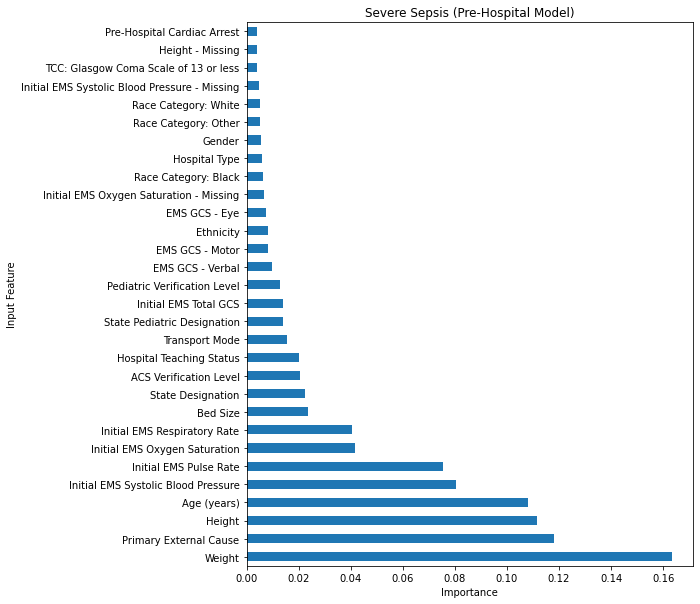

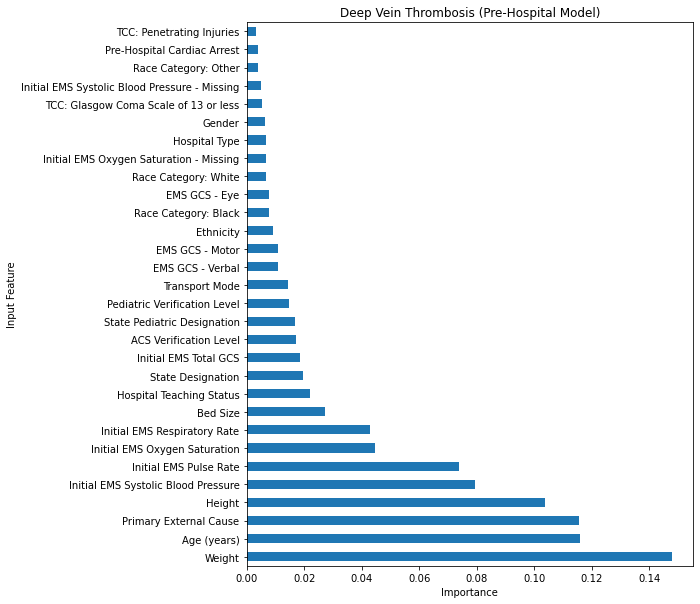

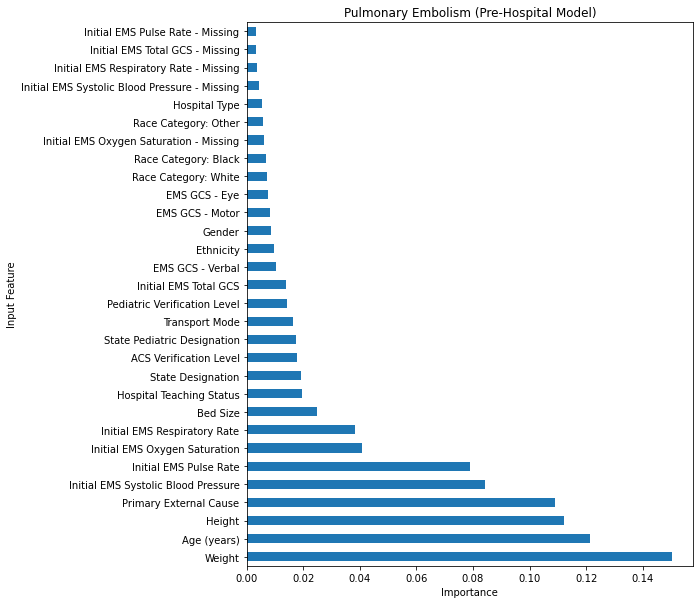

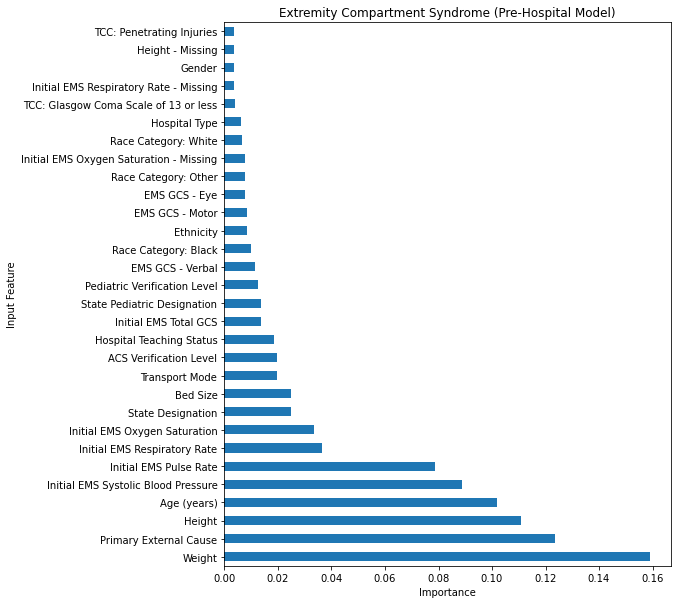


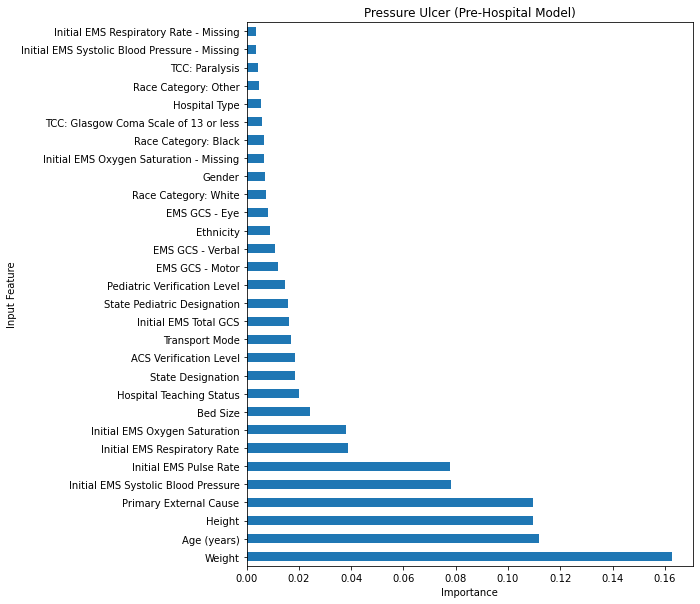

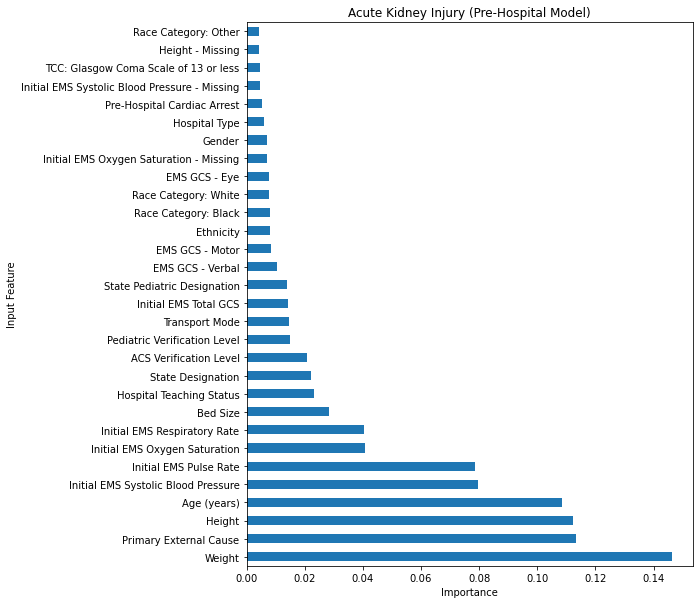


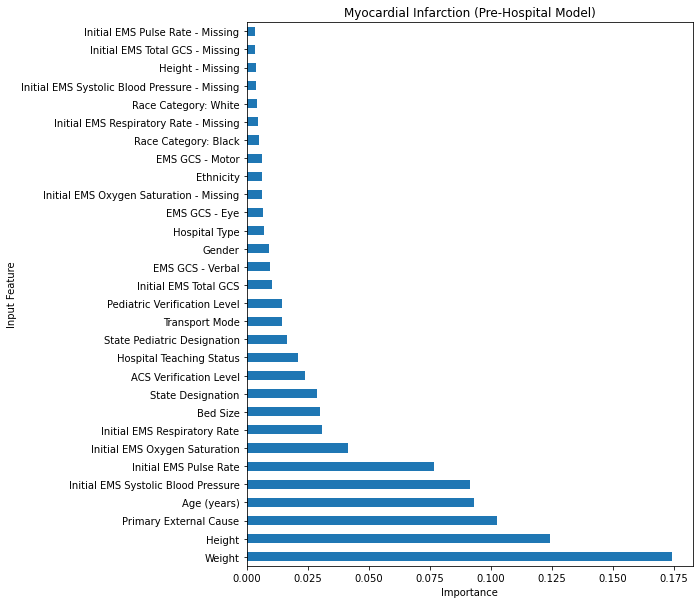

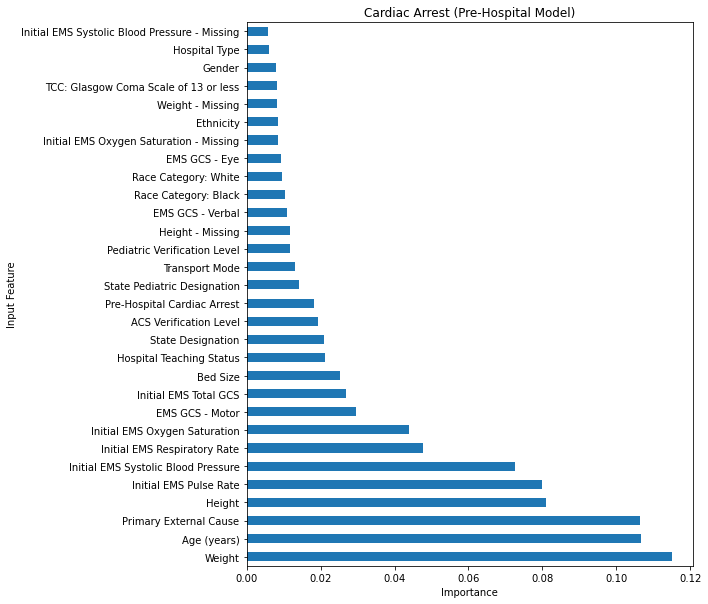


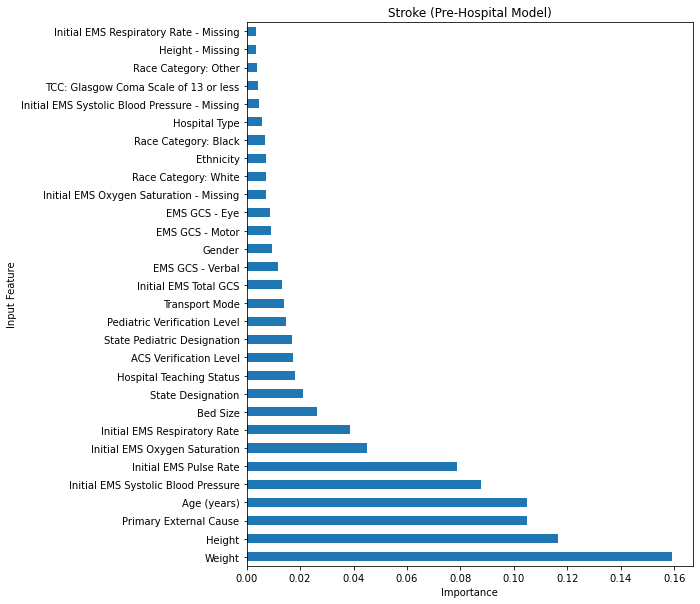

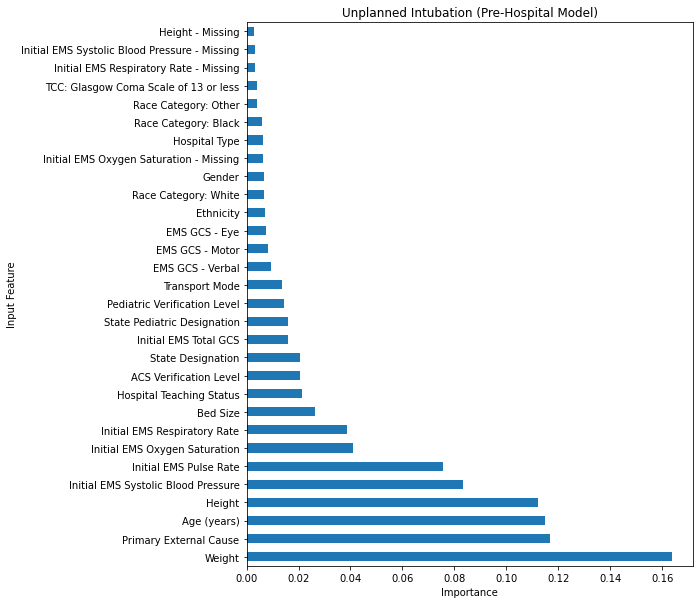


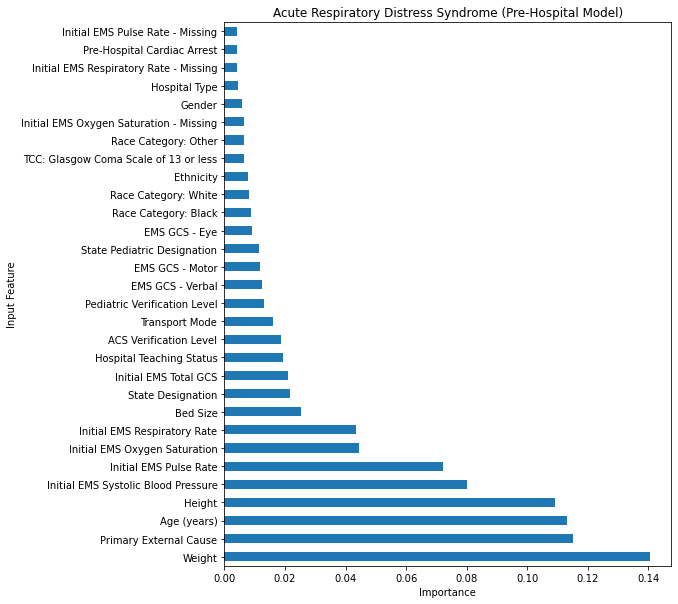

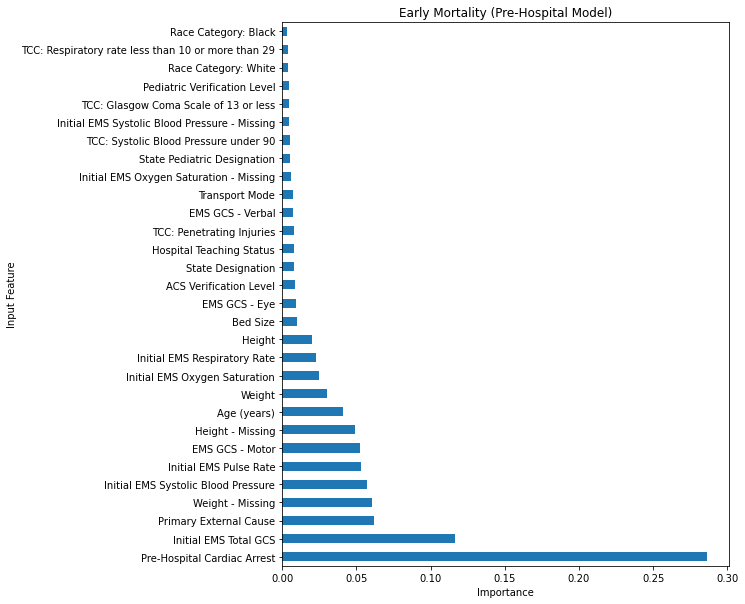


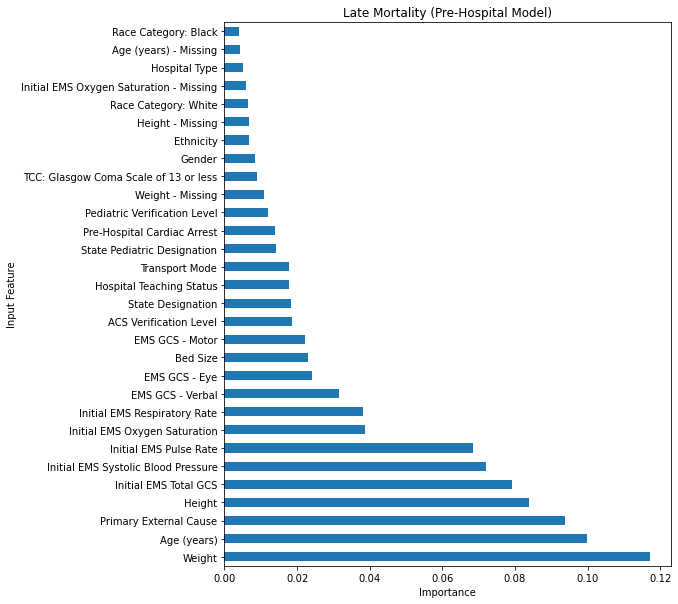


**Supplementary Figure 1B.** Random Forest Feature Importance – ED Model


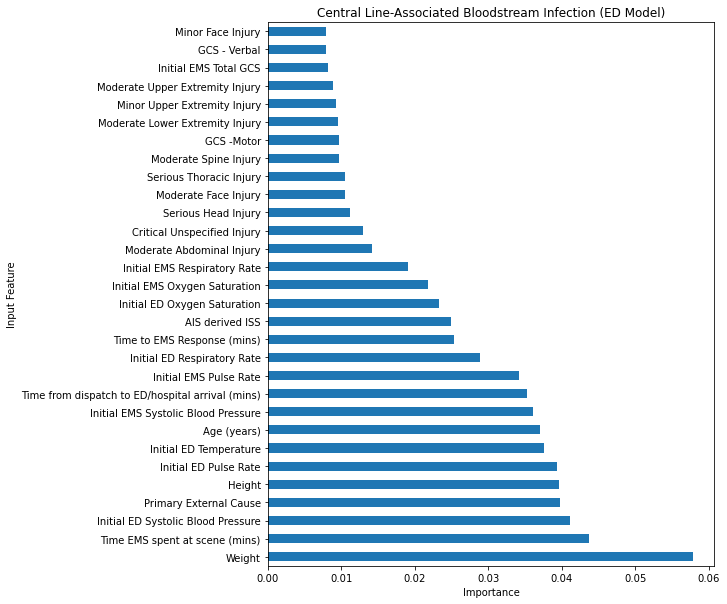

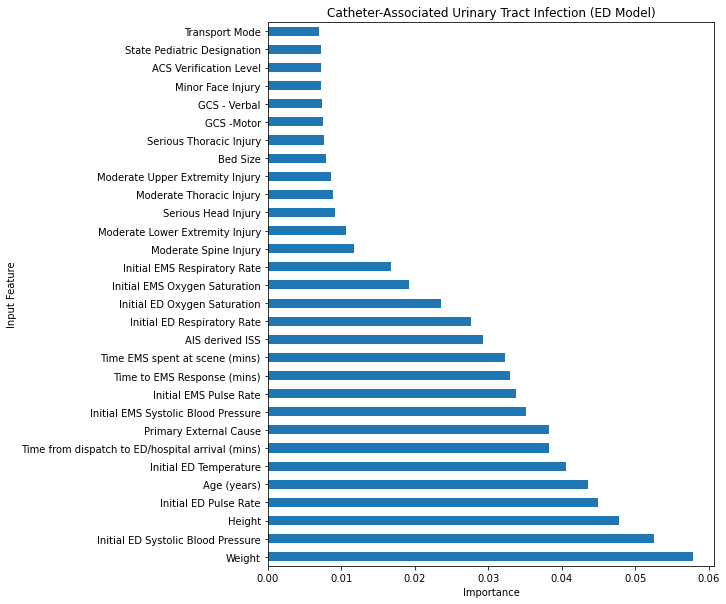

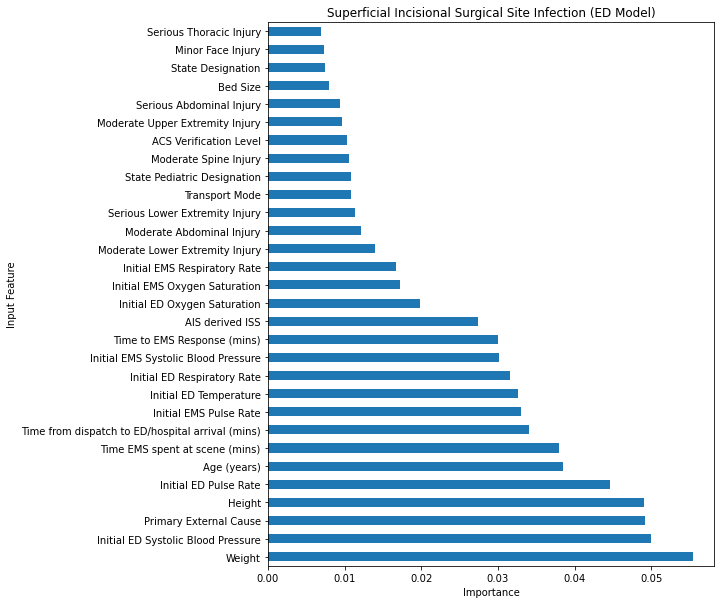

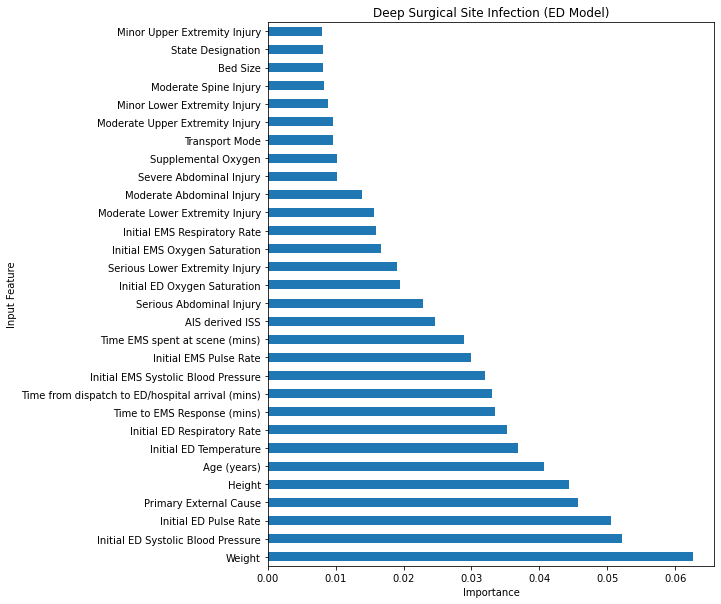

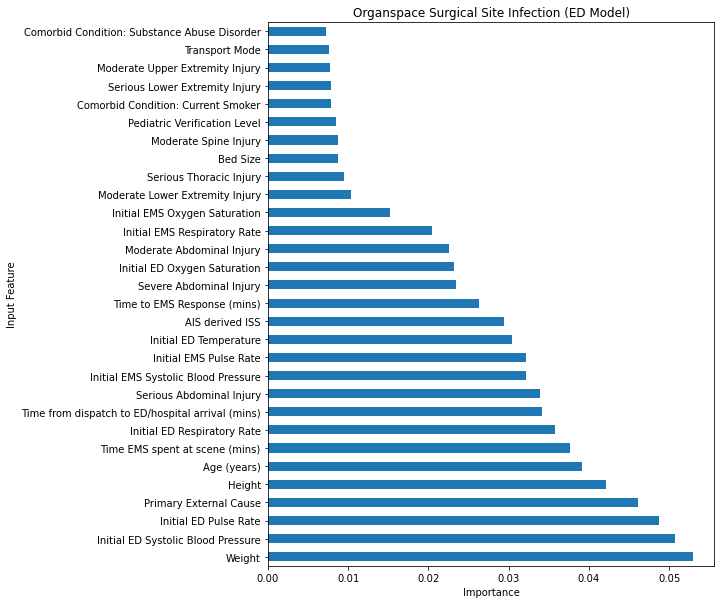

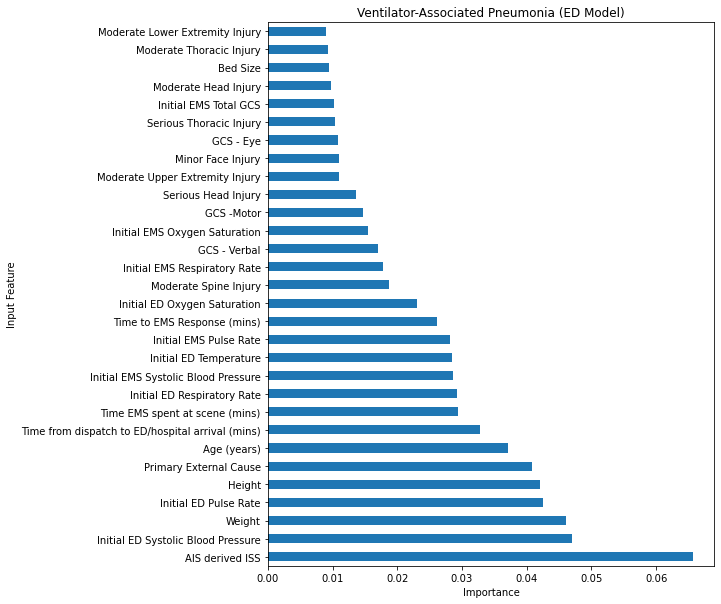

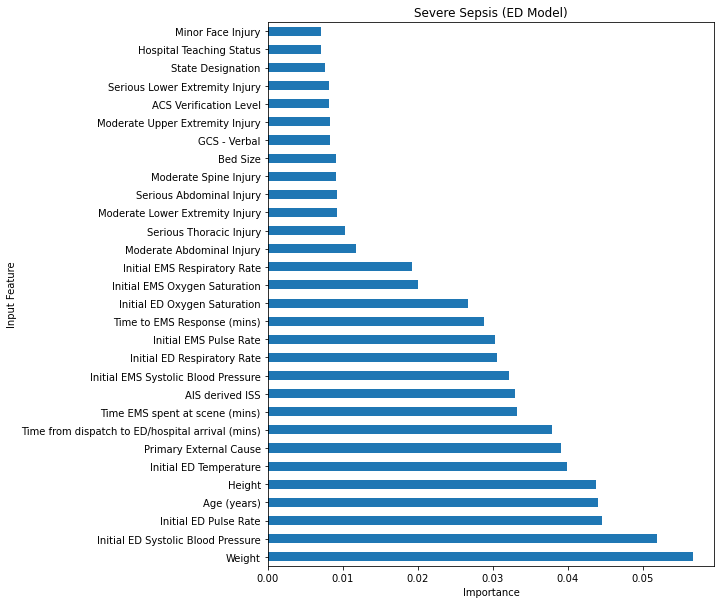

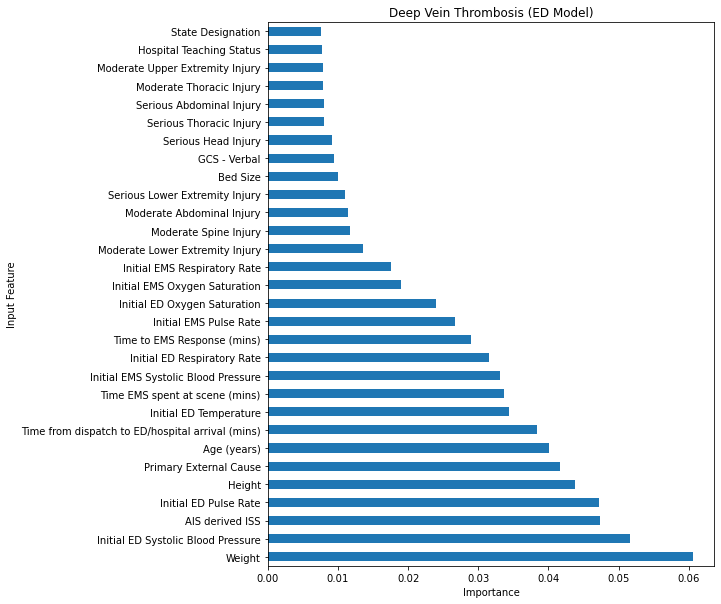

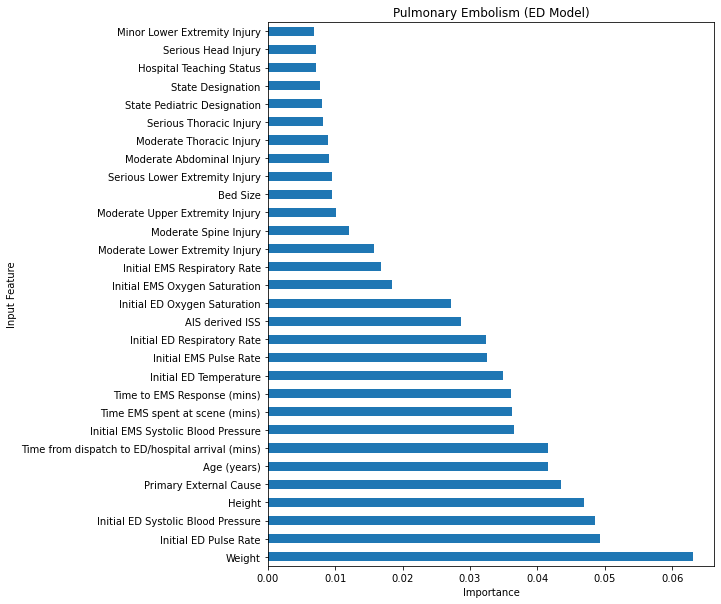

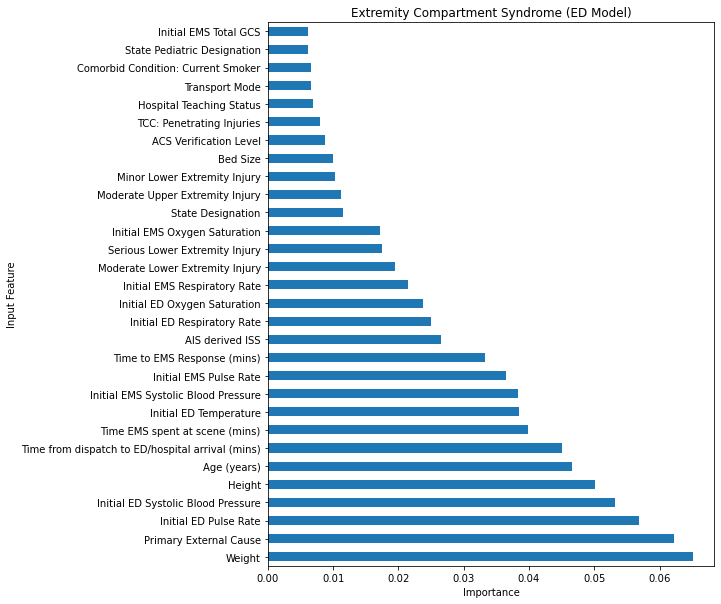

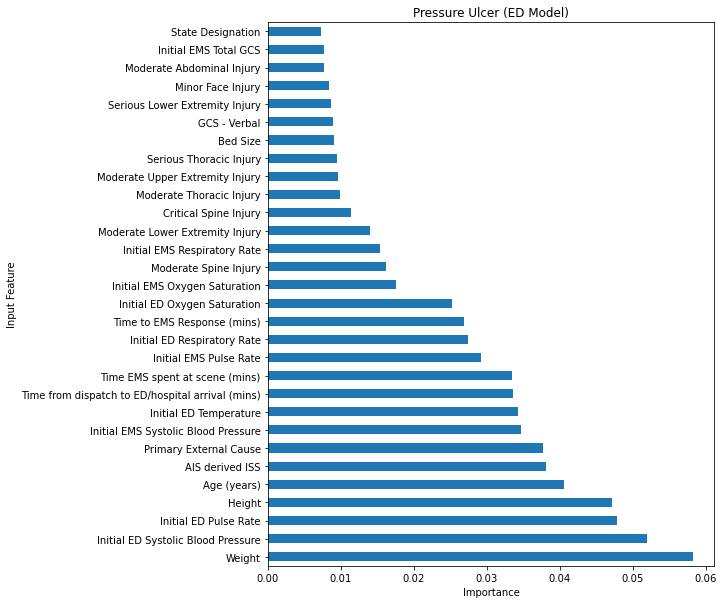

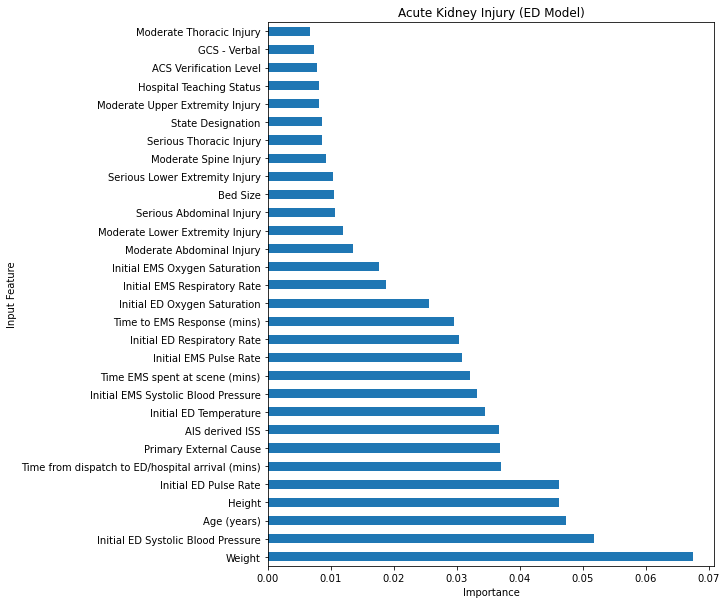

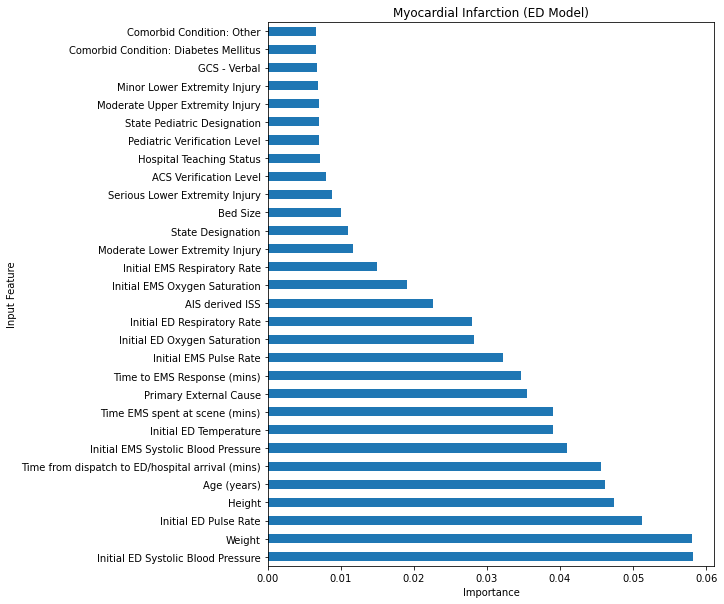

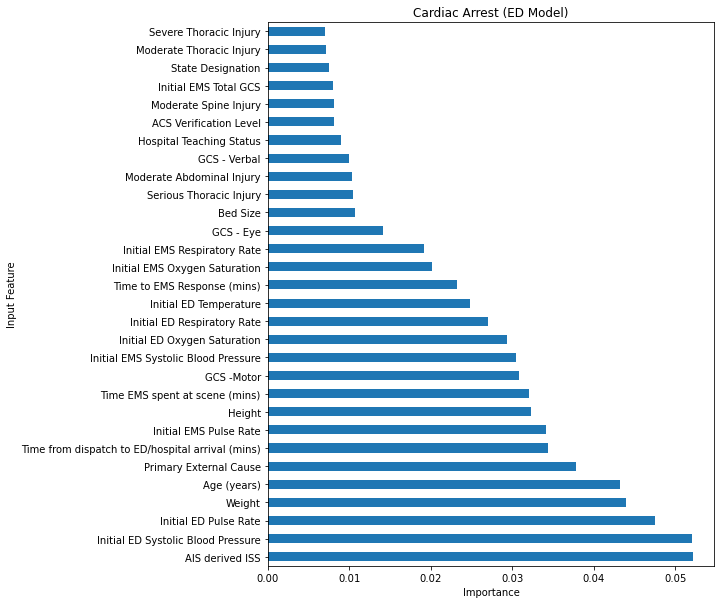

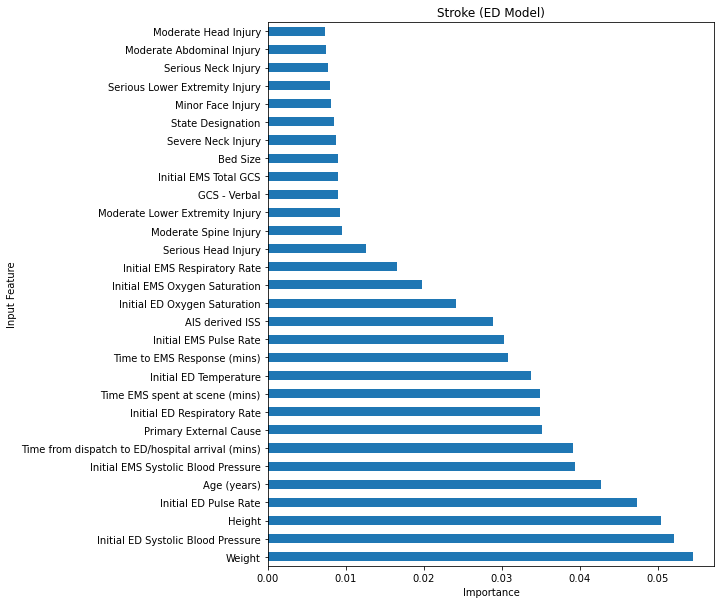

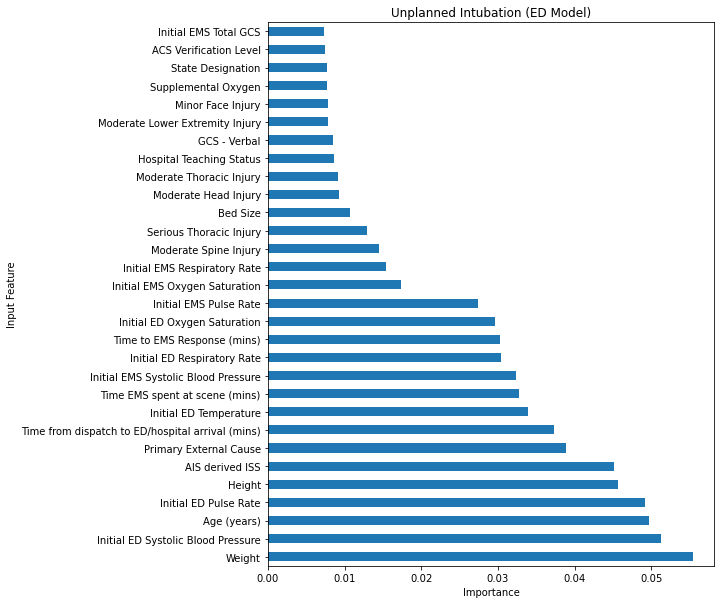


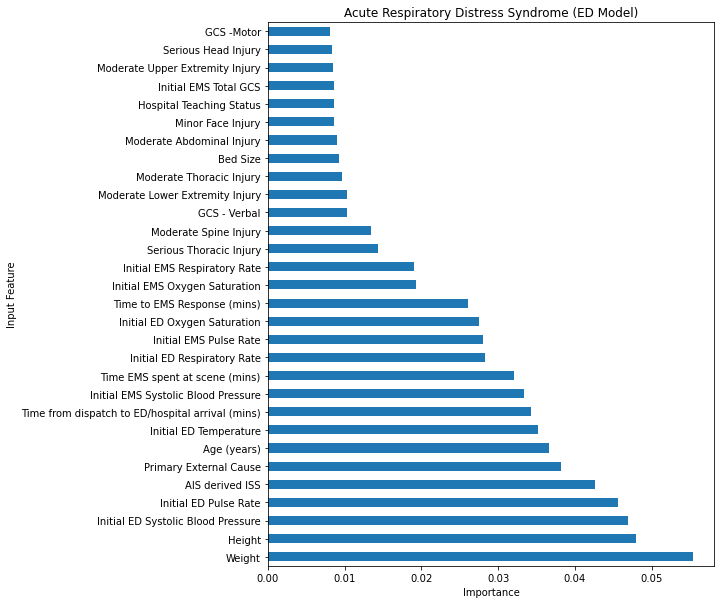

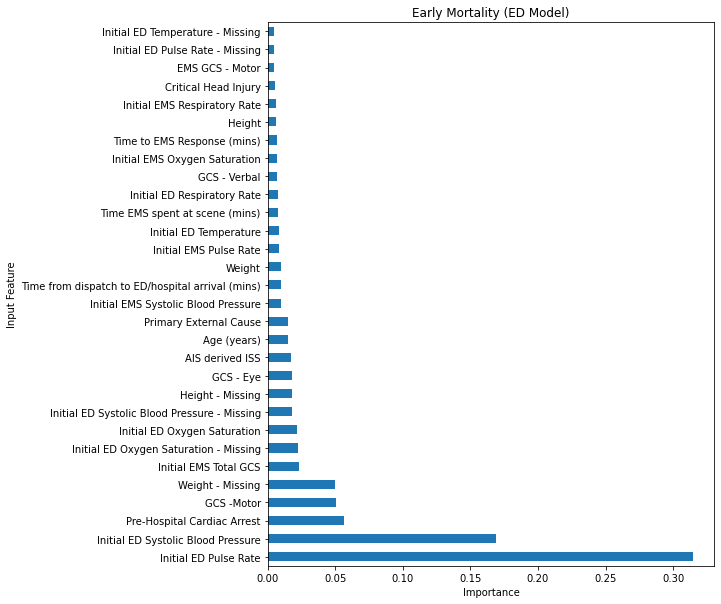


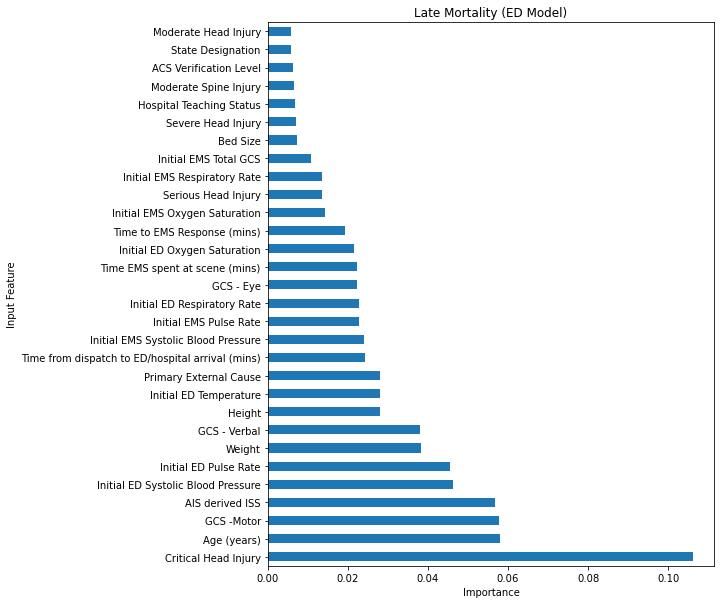


**Supplementary Figure 1C.** Random Forest Feature Importance – In-Hospital Model


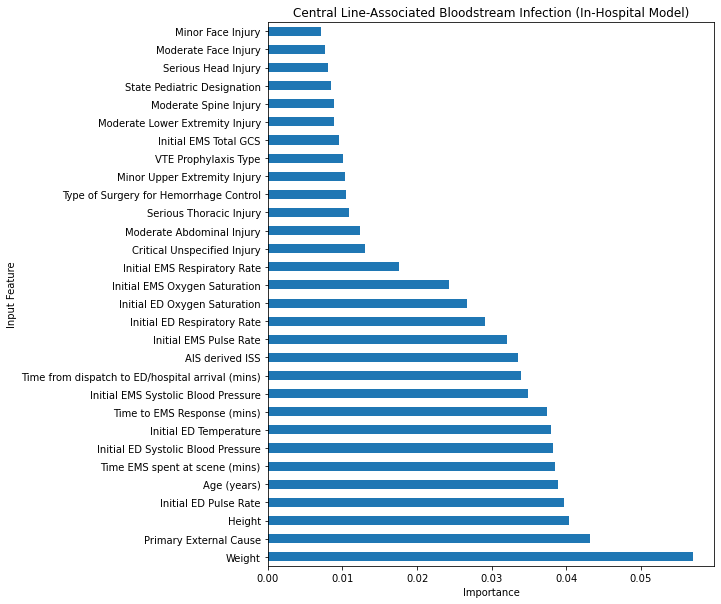

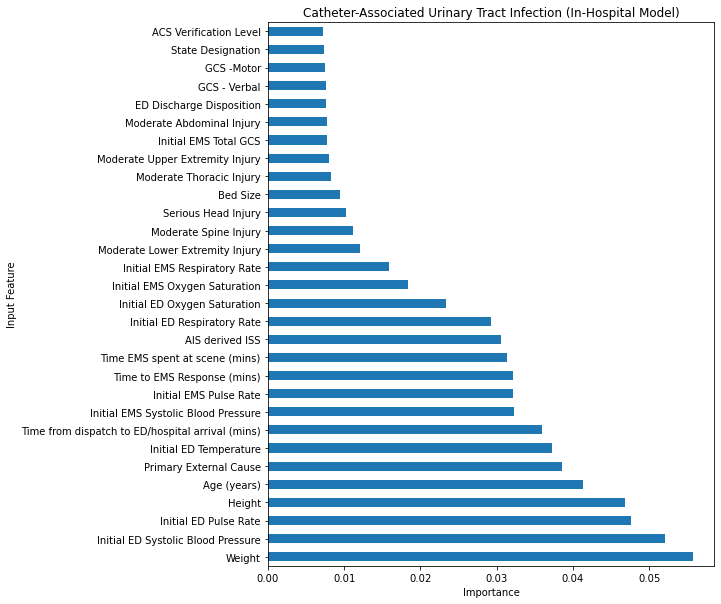

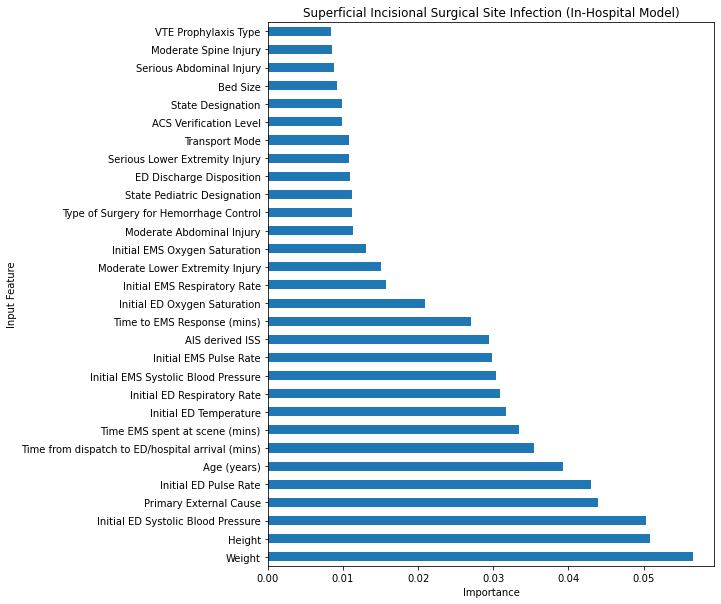

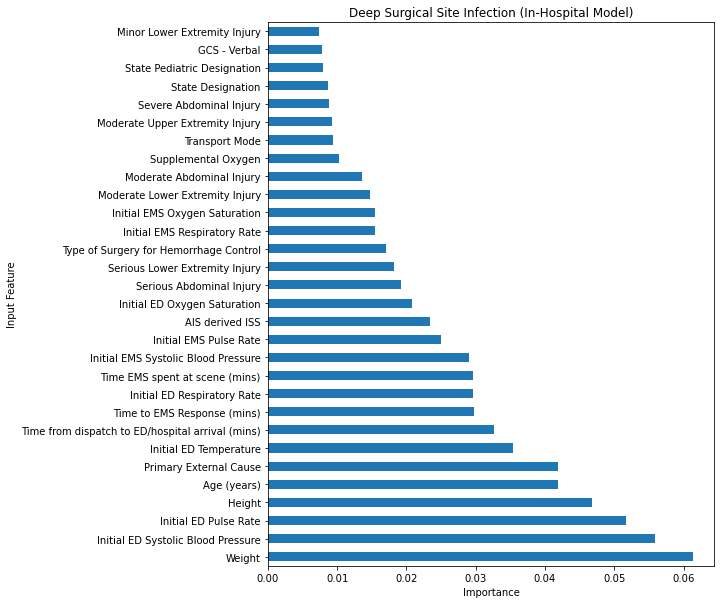

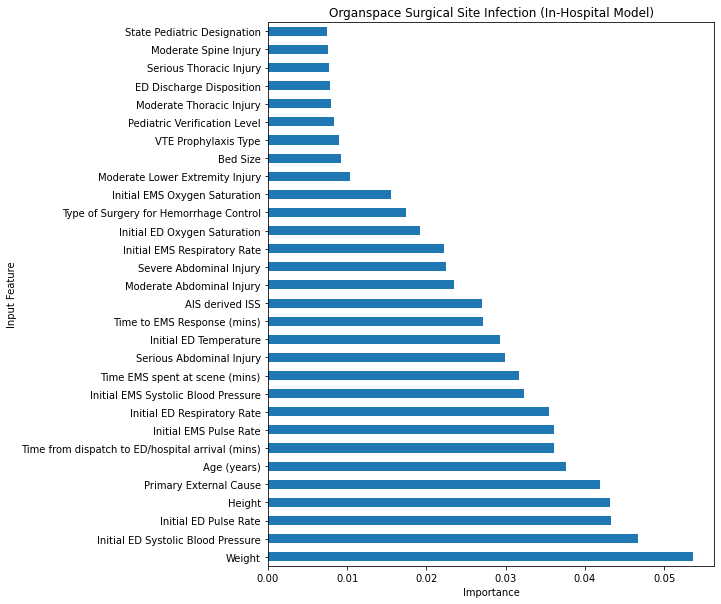

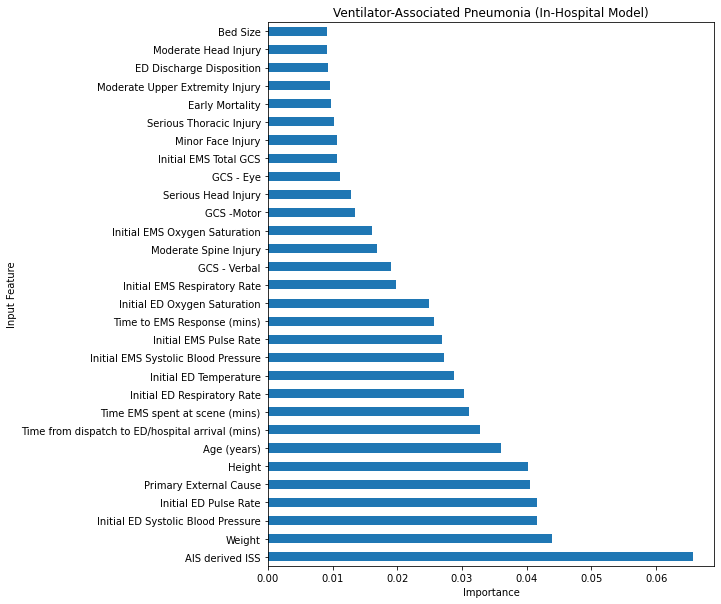

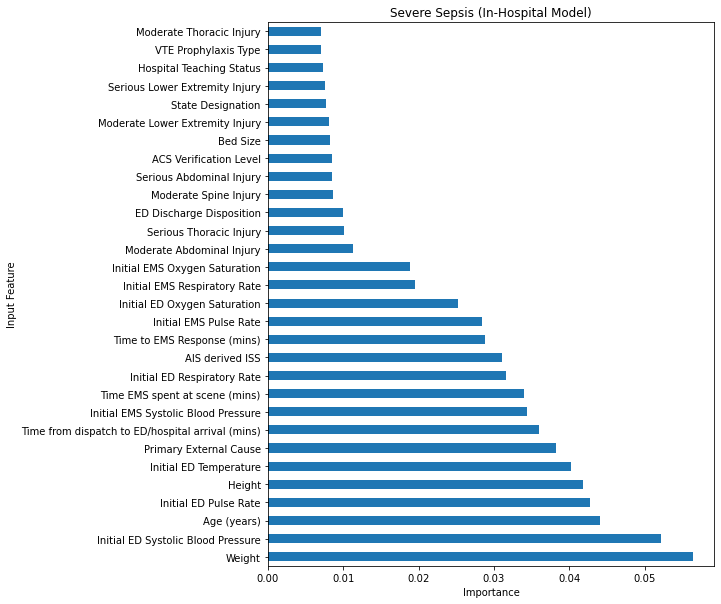

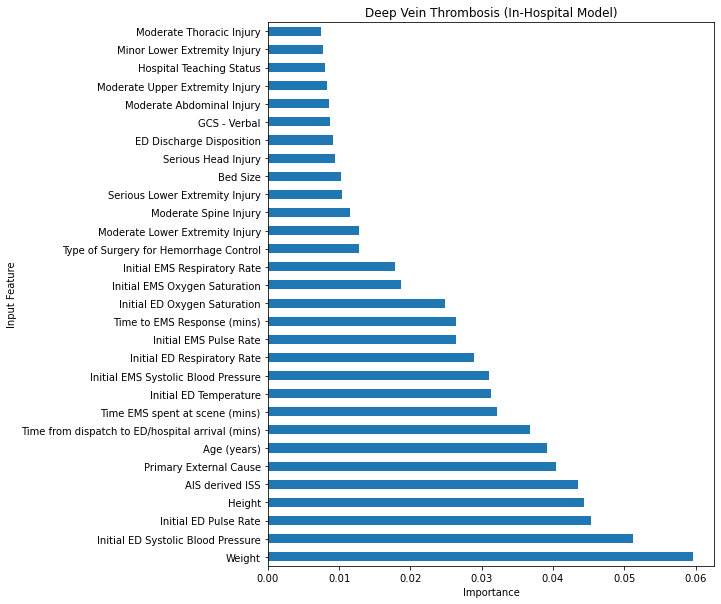

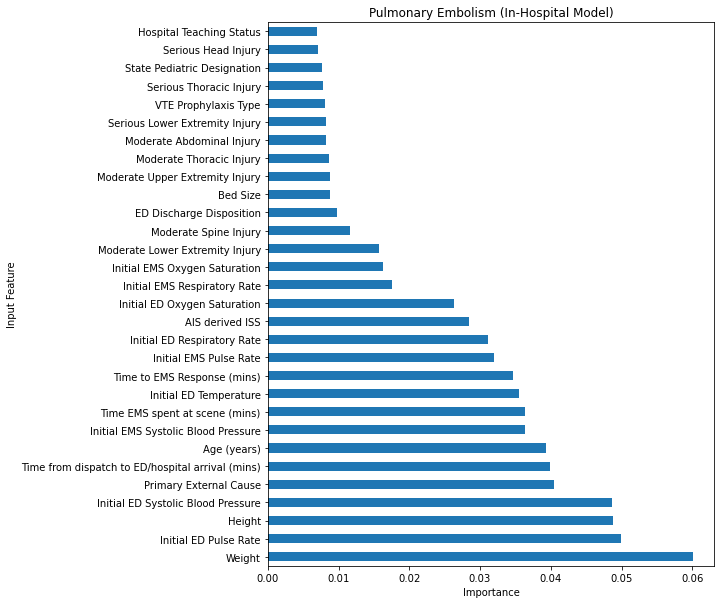

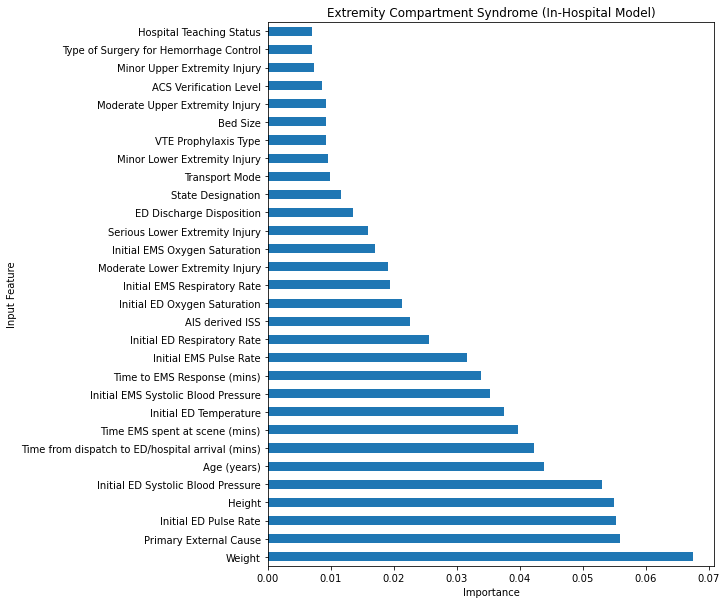

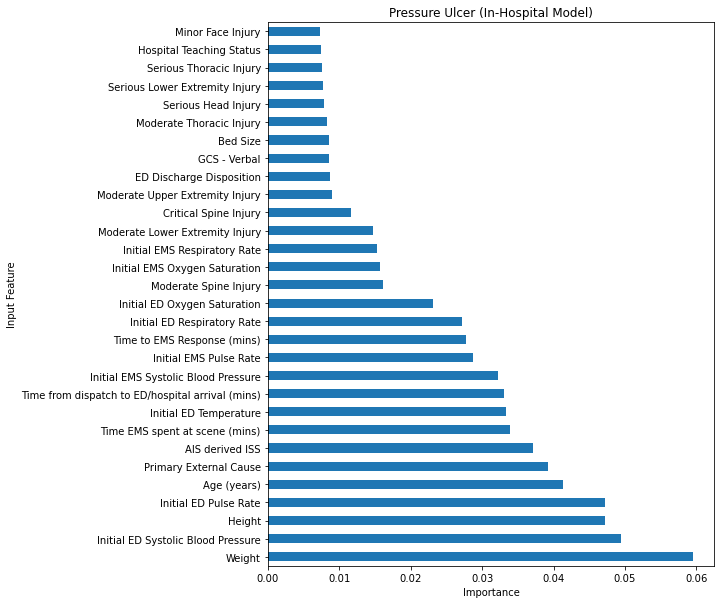

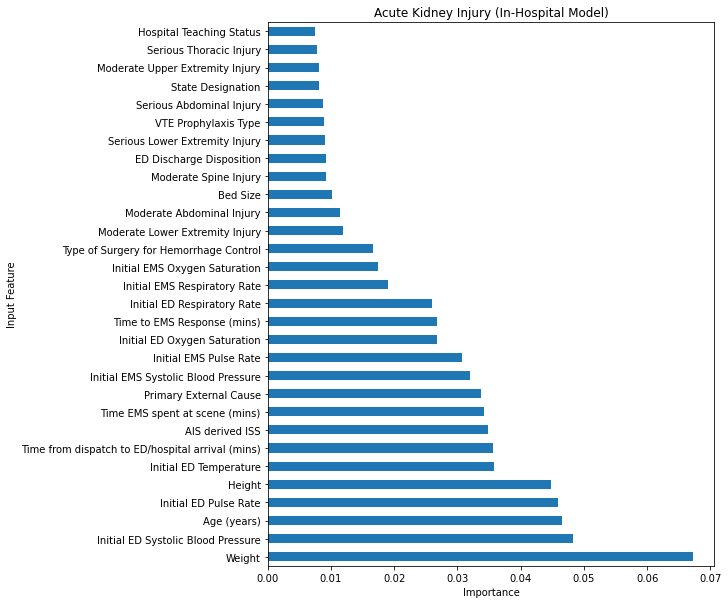

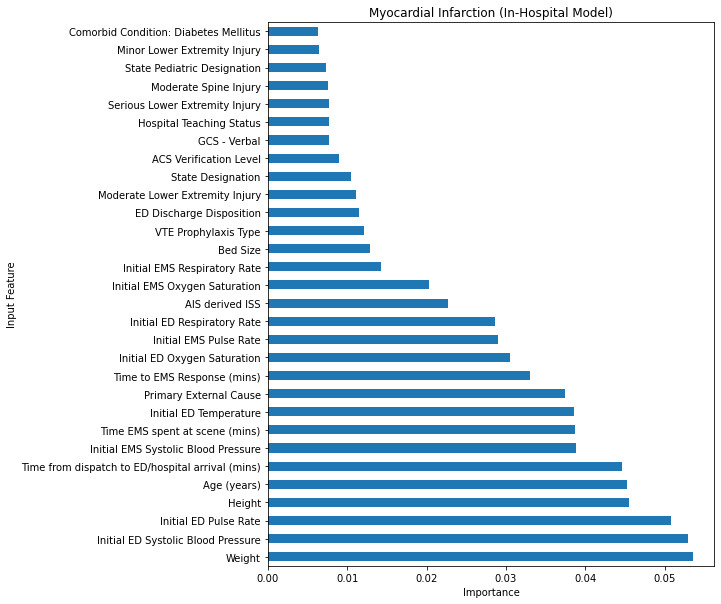

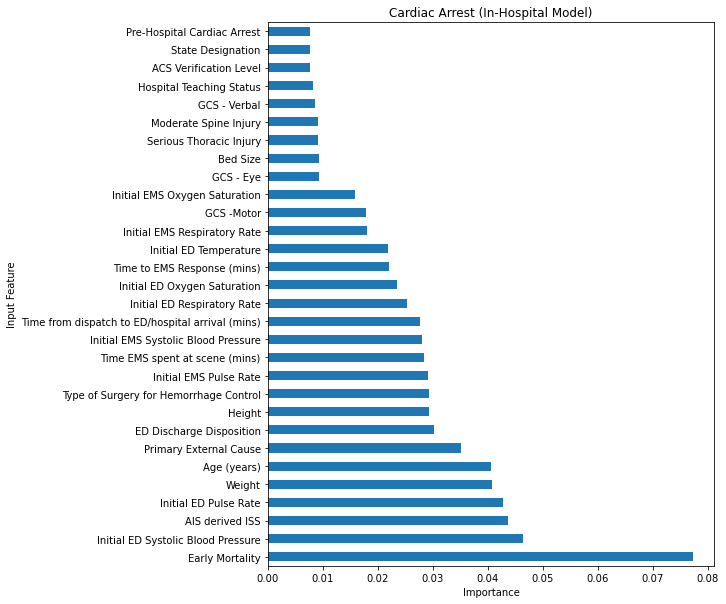

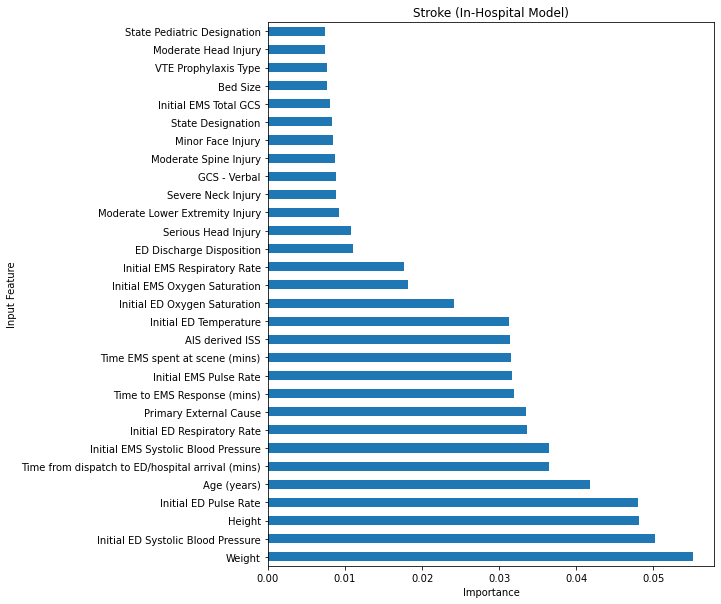

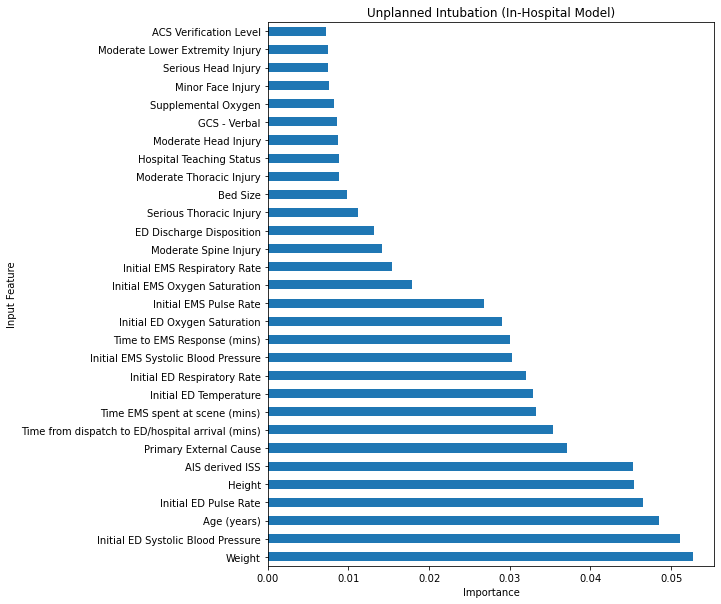

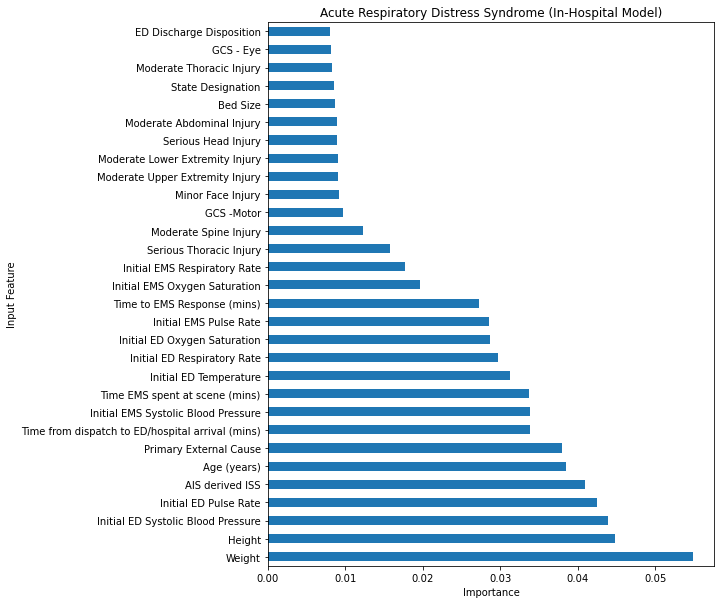

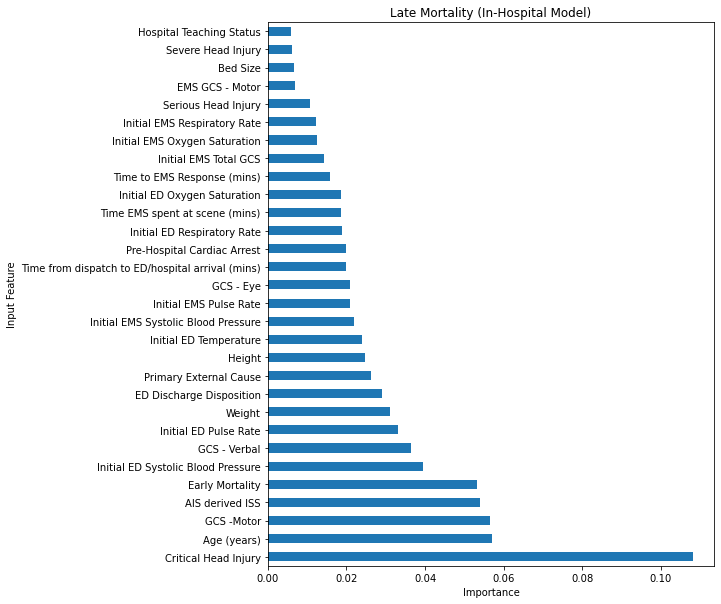

Supplement: Supplementary file 1 — Supplementary Information. [file 41598_2023_32453_MOESM1_ESM.docx]
